# RelA Signaling in *Scgb1a1+* Progenitors Mediates Lower Airway Epithelial Atypia in RSV-Induced Post-Viral Lung Disease

**DOI:** 10.3390/ijms27062864

**Published:** 2026-03-21

**Authors:** Melissa Skibba, Allan R. Brasier

**Affiliations:** 1 Department of Medicine, School of Medicine and Public Health, University of Wisconsin-Madison, Madison, WI 53705, USA; skibba2@wisc.edu; 2 Institute for Clinical and Translational Research, University of Wisconsin-Madison, Madison, WI 53705, USA

**Keywords:** post-viral lung disease, club cell progenitors, alveolar regeneration

## Abstract

Respiratory syncytial virus (RSV), a member of the genus *Orthopneumovirus*, is an etiological agent in infant lower respiratory tract infections (LRTIs) producing substantial global morbidity. Here, secretoglobin (*Scgb1a1*)-derived progenitors play a primary role in triggering innate, inflammatory, and cell state transitions in response to RSV LRTIs. Whether RSV activation of innate signaling in this epithelial sentinel population leads to chronic airway disease is unknown. To understand the role of innate signaling in *Scgb1a1*-derived progenitors, a model of RSV post-viral disease (PVLD) was developed and studied in the presence or absence of RelA conditional knockout (CKO). Single-cell RNA sequencing (scRNA-seq) studies showed that RSV-PVLD induced a transition of atypical, differentiation-intermediate, alveolar type 2 (aAT2) cells characterized by tumor protein 63 (TRP63), aquaporin 3 (AQP3), and Itgβ4 expression, as well as changes in PDGFRβ mesenchyme. A single-cell trajectory analysis and lineage-tracing experiments using *Scgb1a1* CreER^TM^ X mTmG mice demonstrated that the *Scgb1a1+* populations were precursors to the aAT2 population. Mechanistically, we found that the formation of the aAT2 population was prevented by RelA CKO. A differential gene expression analysis revealed that RSV-PVLD coordinately upregulates nuclear receptor subfamily 1 group D (*Nr1d1/2*), clock and basic helix-loop-helix ARNT-like 1 (*Bmal*) genes both in the aAT2 cell and in its *Pdgfrα*+ mesenchymal niche in a RelA-dependent manner. A systematic analysis of intercellular epithelial–mesenchymal communication in the scRNA-seq data showed that the clock-dysregulated epithelial–mesenchymal niche produces aberrant *ANGPTL4* expression. ANGPTL4 upregulation was confirmed by the measurement of both its mRNA and protein. Moreover, ANGPTL4 is biologically active in the BALF of RSV-PVLD mice, inhibiting lipoprotein lipase activity. We conclude that RSV-PVLD is mediated, at least in part, by RelA signaling in *Scgb1a1*-derived epithelial progenitors, dysregulating ANGPTL4 signaling in an epithelial–mesenchymal niche, resulting in persistence of atypical alveolar epithelial cells with dysregulated of clock gene expression.

## 1. Introduction

The *Orthopneumovirus*, respiratory syncytial virus (RSV), is an etiological agent in infant lower respiratory tract infections (LRTIs), producing substantial morbidity globally [1] and representing the most common cause of pediatric hospitalizations [2]. More importantly, children hospitalized with RSV LRTI exhibit persistent reductions in lung function, utilize health care resources at greater rates [3,4,5,6], exhibit persistent obstructive airway disease as young adults [7], and are at a two-fold increased risk of premature death from a respiratory disease [5]. Interpreted with the findings that post-LRTI show a greater incidence of aeroallergen sensitization [3], these findings suggest early life RSV LRTI is associated with structural and immunological remodeling of the airways.

Upon initial infection, RSV replicates throughout the epithelium; as the RSV disseminates into the lower airway epithelium, its replication produces mucosal injury, barrier disruption, and the activation of innate immunity, triggering remodeling pathways [8,9,10]. Because high levels of RSV replication are associated with enhanced disease severity and longer hospitalization in naïve infants [11,12], innate responses of small airway epithelial cells play a critical role in determining the disease outcome [13,14,15,16,17,18]. However, the role of the epithelial innate immunity in determining long-term outcomes of RSV LRTI is not fully understood.

In infections involving the lower airway, RSV replication produces ciliated cell sloughing, resulting in disruption of the epithelial surface [9], activating progenitor cell populations to initiate repair. Depending on the location and severity of injury, progenitor cells arise from distinct mesenchymal niches. In the terminal alveoli, alveolar type (AT2) stem cells normally repopulate the AT1, but this population does not repopulate the distal bronchioles [19]. Studies using models of distal small airway injury have identified distinct progenitor populations in the parenchyma that are recruited to expand and repopulate the lower airway and alveoli. These progenitors are derived from a population of secretoglobin (Scgb1a1)-expressing basal cells, primarily located within the broncho-alveolar duct junction, where they can contribute to ~50% of the distal alveolar population [20]. Spatial transcriptomics and single-cell RNA-seq (scRNA-seq) studies have found that *Scgb1a1+* progenitor cells are maintained in mesenchymal niches engaged in trophic interactions with platelet-derived growth factor receptor (PDGFR)α-expressing fibroblasts [21]. These mesenchymal interactions not only maintain the “stemness” of progenitor cells but also participate in their expansion in response to injury [21,22]. The identity and source of progenitor epithelial populations in RSV-induced lung injury are not known.

Previously, we found that the *Scgb1a1*-expressing progenitor lineage is one of the major sentinel cells that initiate inflammatory responses to RSV infections in the lower airways [23,24]. Here, intracellular RSV replication activates the NFκB/RelA pathway, a pathway that induces secretion of Th2-polarizing chemokines, type I/III interferons (IFN), and mesenchymal growth factors [25,26,27,28,29,30,31]. RelA conditional knockout (CKO) in *Scgb1a1* progenitors show significantly reduced neutrophilic inflammation, epithelial-dependent chemokine expression, and myofibroblast expansion in response to RSV [23]. These data place RelA signaling in *Scgb1a1* progenitors as central to a coordinated innate and inflammatory response to lower airway viral infection. The role of *Scgb1a1* progenitors in RSV infection-induced repair and remodeling has not been explored.

Viral infections prior to adult epithelial differentiation produce distinct structural remodeling responses known as post-viral lung disease (PVLD) [32,33,34]. Reasoning that RelA signaling in the *Scgb1a1* progenitor cell may play a role in epithelial remodeling in RSV-PVLD, we established a model of RSV-PVLD and investigated the effect of RelA CKO in a mouse model. scRNA-seq was utilized, which shows that RSV induces an accumulation of atypical alveolar type 2 (aAT2) cells expressing the tumor-related protein (TRP63+) and integrin (ITG)a6b4, characteristic of differentiation-intermediate transitional cells [20]. Furthermore, lineage-tracing of *Scgb1a1*-derived progenitors using GFP tagging shows that RSV infection induces this population to appear in the alveolar parenchyma that express TRP63+ cells and exhibit persistent RSV glycoprotein expression. The differential gene expression analysis confirmed that RSV-PVLD disrupts an aAT2 epithelial mesenchymal niche through the upregulation of inflammatory clock genes. Using probabilistic cell–cell communication inference, we infer that RSV disrupts trophic mesenchymal–epithelial signaling pathways, including the angiopoietin-like (ANGPTL) pathway. This data provides new mechanistic insights into the role of innate epithelial signaling in RSV-PVLD by identifying the *Scgb1a1* progenitor as a progenitor cell arising from within a dysregulated epithelial–mesenchymal niche.

## 2. Results

### 2.1. RSV Post-Viral Lung Disease (PVLD)

**Figure 1:**
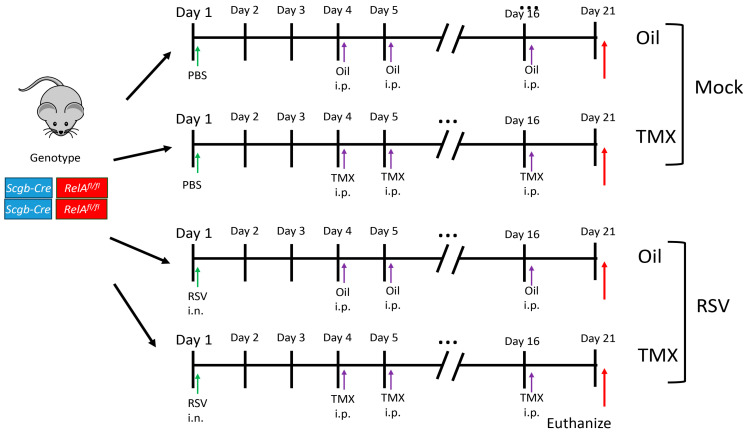
RSV post-viral lung disease (PVLD). The shematic diagram of the RSV-PVLD model. Three five-week-old *Scgb1a1*-CreER^TM^ X RelA^fl/fl^ mice (both sexes) in the C57BL6/J background are mock-treated or infected via the i.n. route. Three days after the infection, the mice are randomized (arrows) to receive corn oil or tamoxifen (TMX) treatments daily via i.p. until d16. At 21 d post-infection, the mice were euthanized for analyses.

To explore the chronic effects of post-RSV remodeling and determine the mechanistic role of epithelial intrinsic immunity on progenitor expansion in RSV LRTI, we established a PVLD model, where immature (3-week-old) homozygous for *Scgb1a1* Cre recombinase (CreER^TM^) X RelA^fl/fl^ mice in the C57BL6/J background [25,26] were mock-treated (PBS) or infected with RSV (Figure 1). The mice were then randomized to vehicle (corn oil) or tamoxifen (TMX) treatment, the latter to induce RelA CKO after the resolution of acute infection (for simplicity in the following section, we refer to corn oil-treated mice as “WT mice” and TMX-treated mice as “RelA CKO”).

### 2.2. TMX Induces an Effective Knockdown of the RelA Pathway in RSV-PVLD

**Figure 2:**
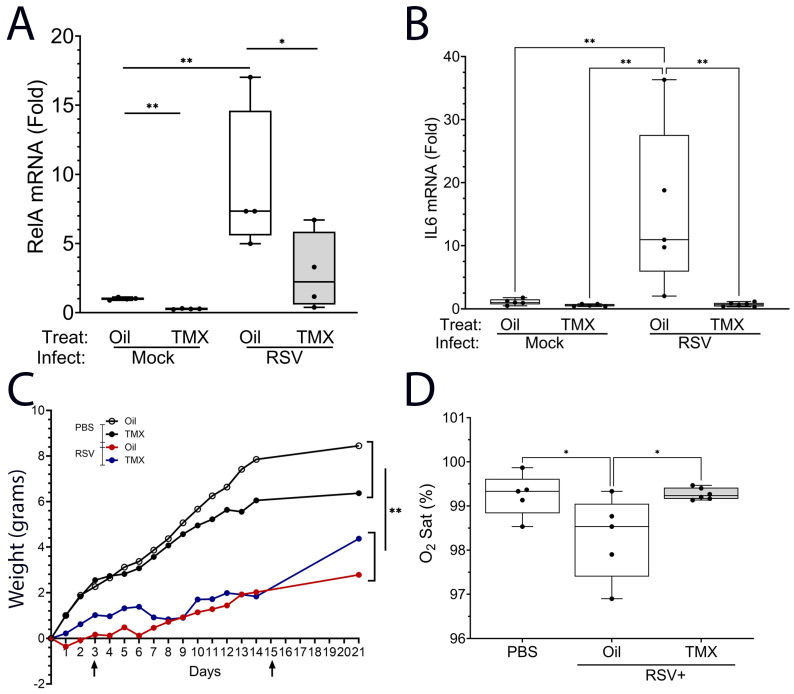
Inducible RelA CKO. Total lung RNA was extracted from RSV-PVLD at 21 d. RNA expression is shown as fold change normalized to *Gadph* as internal control. (**A**) RelA mRNA; (**B**) IL6 mRNA RSV. A 25–75% interquartile range (IQR) is shown; each symbol is an independent animal (n = 5–6). * *p* < 0.05; ** *p* < 0.01, post hoc. (**C**) Weight difference for treatment groups over time (days). Vertical arrows, timing of TMX treatment. ** *p* < 0.01. (**D**) Resting oxygen saturation by treatment group (n = 5 for each group). A 25–75% IQR is shown. * *p* < 0.05.

We first sought to confirm that RelA CKO was successful by measuring RelA and its downstream pathway. In the TMX-treated mice, *RelA* mRNA expression was reduced to 22% that of Ool-treated mice in the absence of infection (0.22 ± 0.11 vs. 1 ± 1.1-fold; *p* < 0.01, number (n) = 5; Figure 2A). By contrast, RSV infection induced *RelA* mRNA by 9.1 ± 5.3-fold in WT mice, an increase that was significantly reduced by TMX treatment to 2.9 ± 2.8-fold, confirming the effect of Cre recombination on the target *RelA* gene (n = 5, *p* < 0.05; Figure 2A). To confirm the impact of Rel CKO on its downstream pathway, we measured the expression of interleukin 6 (IL6), a RSV-inducible epithelial derived cytokine that is directly activated by RelA. Here, we observed that RSV induced a 15.6 ± 13-fold-fold increase in *IL6* mRNA (n = 4, *p* < 0.001, Figure 2B), confirming the activation of the RelA pathway. Strikingly, the TMX treatment reduced IL6 mRNA expression to 0.67 ± 0.3-fold (n = 4, *p* < 0.01, Figure 2B). These data confirm the effective inducible inhibition of *RelA* and its downstream pathway in this model.

Over the 21-day course of the experiment, uninfected mice gained ~4–6 gm in body weight, irrespective of the WT or Rel CKO status (ns, Figure 2C). By contrast, RSV-infected mice gained an average of 3 gm, also irrespective of the WT or Rel CKO status (Figure 2C). This difference in weight was only significant between the mock-infected and RSV-infected groups (*p* < 0.05, Figure 2C).

To establish whether RSV affected pulmonary gas exchange, resting O_2_ saturation was measured by collar oximeter. In the mock-infected WT mice, ambient O_2_ saturation (Sat) was 99 ± 0.3%, whereas O_2_ Sat in RSV-PVLD in WT mice was significantly reduced, to 97 ± 0.3%, and RelA CKO restored ambient O_2_ saturation to 99 ± 0.1% (*p* < 0.01, n = 5–6, Figure 2D). The difference in O_2_ Sat was seen in both sexes (Supplementary Figure S1).

### 2.3. RSV mRNA Detection and the Analysis of Replication

**Figure 3:**
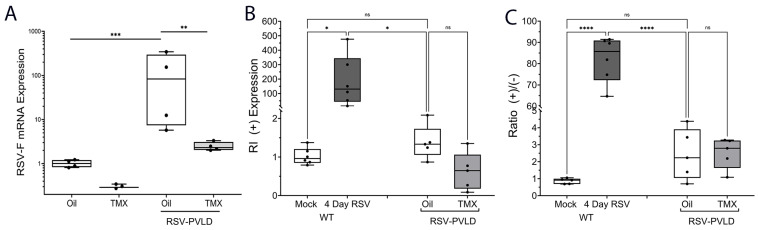
Strand-specific RSV replication analysis. (**A**) Positive-sense RSV mRNA quantititation. RSV F mRNA expression is shown as fold change normalized to *Gadph* as internal control. A 25–75% interquartile range (IQR) is shown; each symbol is an independent animal (n = 5–6). ** *p* < 0.01; *** *p* < 0.001, post hoc. (**B**), Positive-strand quantitation of RSV replication intermediate (RI). Positive controls are WT C57BL6/J mice acutely infected with RSV for 4 d (n = 6). * *p* < 0.05; ns, not significant; post hoc. Note the >200 fold induction of RI in acute infection that declines to levels indistinguishable from mock infected mice. (**C**) The ratio of positive/negative RSV genome. **** *p* < 0.0001; ns, not significant. Note presence of a high (+)/(−) ratio at d4, consistent with active RSV replication that wanes by d21 in the RSV-PVLD model.

We also observed that RSV F mRNA was persistently detectable in RSV-infected RelA WT mice, with an induction of 129 ± 158-fold relative to mock-treatment (Figure 3A). Interestingly, this level of RSV mRNA was reduced in RSV-infected RelA CKO mice (Figure 3A). In a manner consistent with many other studies in the mouse model, we were unable to detect infectious RSV plaques on d21 in the RSV-PVLD BALF. However, this insensitive assay may not be able to detect the presence of low levels of RSV replication [35]. To further understand this finding, we explored the possibility of persistent RSV replication by applying an established, strand-specific Q-RT-PCR assay, capable of detecting the presence of replicative intermediate (RI) RSV genomic RNA [35]. Here, positive-sense RNA is detected using strand-selective cDNA synthesis. Using this approach, we found that the positive strand was highly abundant in the lungs of the mice during acute RSV infection (at 4 d, Figure 3B) and that the positive/negative strand ratio, a reliable indicator of RSV replication, was elevated by ~110-fold, consistent with molecular evidence of RSV replication (Figure 3C). By contrast, the RI abundance in RSV-PVLD was not significantly different from that in mock-infected mice. Moreover, the positive/negative strand abundance was ~2-fold greater, suggesting that there is no molecular evidence of active RSV replication at d21 post-infection in the RSV-PVLD model.

### 2.4. scRNA Sequence Analysis of RSV-PVLD Shows Emergence of Atypical AT2 Populations

To provide greater insight into this coordinated multi-cellular injury repair process, we conducted scRNA-seq of the RSV-PVLD model. Left lungs from n = 4 replicates of each treatment were subjected to 10X Genomics Flex 3’ sRNA sequencing. After QA/QC processing, the libraries were found to have 92.7 ± 0.8% fractions of reads mapped to cells, indicating that the scRNA libraries had negligible ambient RNA contamination (Supplementary Table S3); an integrated dataset of 104K cells X 14.5 K genes was generated. Thirty-five distinct cell types could be resolved based on the expression of unique cell signature markers, including an expansion of atypical AT2-like progenitors.

**Figure 4:**
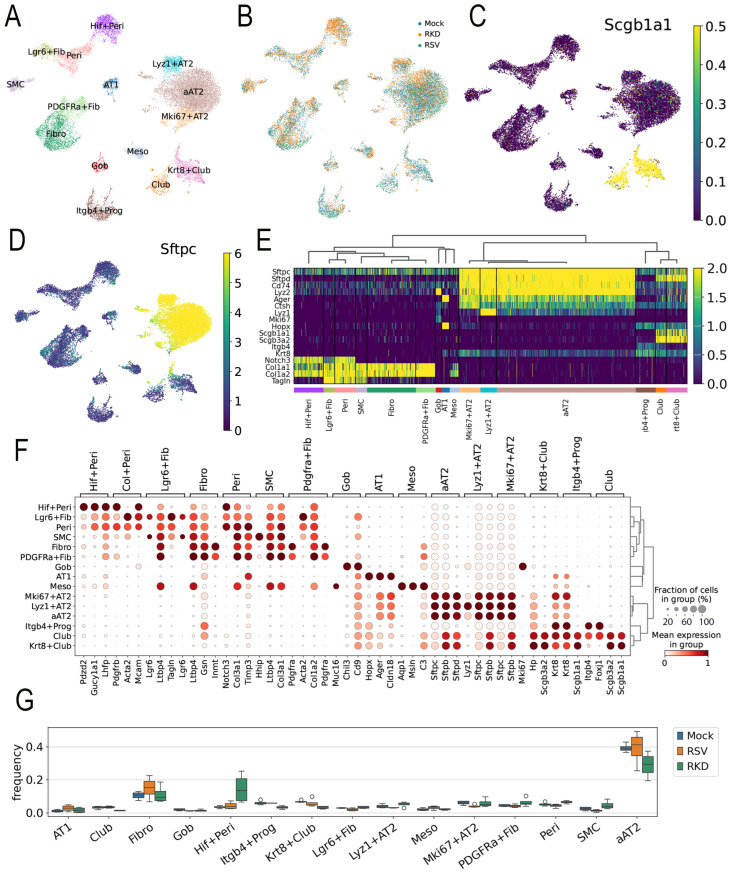
scRNA sequencing analysis. (**A**) Uniform Manifold Approximation and Projection (UMAP) representation of single-cell RNA sequencing of epithelial and mesenchymal cell populations from total lung homogenates from mock-infected, vehicle-treated, RSV-infected, vehicle-treated, or RSV-infected TMX-treated animals harvested 21 d after infection (see Supplementary Figure S3, for a complete spectrum of cell types identified). Each symbol represents a single cell, colored by nearest-neighbor (leiden) clusters and identified by a majority voting of cell markers and unique gene expression modifiers. Abbreviations used: AT, alveolar type; Krt, keratin; Fibro, fibroblast; Fn, fibronectin; Gob, goblet; Hif, hypoxia inducible factor; Lrg, leucine-rich repeat-containing G-protein-coupled receptor; Lyz, lysozyme; Peri, pericyte. (**B**) The UMAP representation of cell types by treatment. Note the presence of distinct populations of aAT2 and Krt8 + club cells induced by RSV in vehicle-treated mice or by RSV with RelA CKO. (**C**) Cell types expressing *Scgb1a1*. Shown is relative expression of *Scgb1a1* mRNA overlaid on the parent UMAP. Note the high *Scgb1a1* expression by the Krt8 + club and club cells. Scale is shown on the right. (**D**) Cell types expressing *Sftpc*. The relative expression of *Sftpc* mRNA overlaid on the parent UMAP is shown. Note the high *Sftpc* expression by the aAT2, *Lyz* + AT2 and *Mki67* + AT2 cells. Scale is shown on the right. (**E**) Heat map of epithelial cell populations for major epithelial cell markers. Abbreviations: Sftp, surfactant protein; Lyz, lysozyme; SDC, syndecan; Hhip, hedgehog interacting protein; Hopx, HOP homeobox, Itgb4, integrin 4; Taglin, transgelin. (**F**) Dot-plot of most highly variable genes for each cell type assignment. Circle diameter refers to the percentage of cell population; color indicates expression level of indicated gene. Note the unique AT2 cell markers surfactant (Sftp)-C and D in the AT2 population, the expression of the AT1 marker, *Ager*, in the same population, and the expression of the progenitor marker *Hopx* in the Krt8 + club cell. (**G**) Cell frequency of epithelial and mesenchymal cell populations by treatment. For each cell type in each treatment, the frequency of cells normalized to total cells was calculated. Mock, mock-infected/vehicle-treated; RSV, RSV-infected/vehicle-treated; RKD, RSV-infected, RelA knockdown (RelA CKO).

Reasoning that the expansion of epithelial progenitors in RSV-PVLD was, in part, due to activation of the mesenchymal–epithelial niche controlling epithelial progenitor expansion, we focused the remaining study on determining the effects of RSV infection on the epithelial–mesenchymal niche. To accomplish this, the data set was filtered for epithelial and mesenchymal cell populations and subjected to leiden nearest-neighbor clustering. A total of 32 transcriptionally distinct cellular populations were assigned based on majority voting of highly expressed markers and manual validation. These groups include a heterogeneous group of surfactant (*Sfpt*)-c+/advanced glycosylation end-product specific receptor (*Ager*) + atypical AT2 (aAT2) cells, lysozyme (*Lyz*)1+ AT2 cells, proliferating *Mki67 +* AT2 cells, keratin 8 (*Krt8*)+/*Scgb1a1*+ club cells, AT1 cells, *Pdgfra*+ fibroblasts, *Lgr6*+ myofibroblasts, *Itgb4*+ progenitors (Prog), and hypoxia factor (*Hif*)1+ pericytes (Peri, Figure 4A). An initial analysis of cell populations affected by RSV was identified by coloring the cells in the UMAP by treatment condition (Figure 4B; individual UMAP plots are shown in Supplementary Figure S2). We noted that RSV infection in the vehicle-treated animals shifted subpopulations in the aAT2, club, *Krt8* + club and *Hif*+ Peri cell groups. RelA CKO affected *Hif* + Peri, *Pdgfra*+ Fibro, and aAT2 populations (Figure 4B).

To identify the cell populations targeted for RelA CKO, *Scgb1a1* mRNA expression was overlaid on the UMAP representation. Here, we observed *Krt8*+ club, and club cells were strongly positive, as expected (Figure 4C). Similarly, aAT2, proliferating (*Mki67*+) AT2 and lysozyme (*Lyz*)1 + AT2 cells were strongly surfactant protein C positive (*Sfptc+*), confirming their identification at AT2 cells (Figure 4D).

Hierarchial clustering of the most highly variable genes was next performed to confirm assigned cellular phenotypes. Here, aAT2, *Mki67* + AT2 and *Lyz* + AT2 cells co-clustered based on shared high levels of expression of the *Sftp* isoforms, including *Sftpc* and *Sftpd* expression (Figure 4E). The aAT2 cluster is a heterogenous collection of AT2 cells with those in intermediate stages of differentiation (Supplementary Figure S4). We term this admixture of cells aAT2 because they co-express *Ager*, the classical cell marker of AT1 cells (Figure 4E). A separate cluster included *Itgb* 4+ progenitors, club- and *Krt8* + club cells that exhibited high levels of *Scgb1a1* and *Scgb3a2* expression (Figure 4E). Also, AT1 cells expressed *Ager* mRNA, as well as the HOP homeobox (*HopX*), a marker of AT2 progenitor responsible for the regeneration of alveoli after hyperoxic injury [36].

A dot-plot clustering of cell types indicated the aAT2, *Krt8*+ club, *Itg*+ progenitor, and club cells had similar transcriptional profiles and grouped separately from the fibroblast and myofibroblast populations (Figure 4F). These data indicated that RSV-PVLD induced aberrant transcriptional profiles of lower airway cells, suggesting intermediate differentiation states.

Next, we examined changes in cell populations using the cell frequency analysis for each cell type by treatment condition. RSV infection induced increases in AT1 and Fib populations but decreased *Lgr6* + Fibro, *Mki67* + AT2 and SMC populations (Figure 4G). By contrast, in the RSV-infected RelA CKO mice, increases in the *Hif*+ Peri and aAT2 cells were observed relative to RSV-infected WT, along with decreases in *Itgb4*+ Prog, *Krt8* + club, and Fib populations (Figure 4G). These data indicate that not only does RSV-PVLD induce shifts in epithelial cell differentiation states, but, unanticipatedly, *Scgb1a1*-directed RelA CKO influences mesenchymal (fibroblast and pericyte) populations. Since the *Lgr6*-expressing fibroblast population is trophic for expansion of *Scgb1a* progenitors [37,38], RSV-PVLD may affect the number or function of these crucial epithelial–mesenchymal niches.

### 2.5. Expansion of Alveolar Progenitor Epithelial Populations in RSV-PVLD

**Figure 5:**
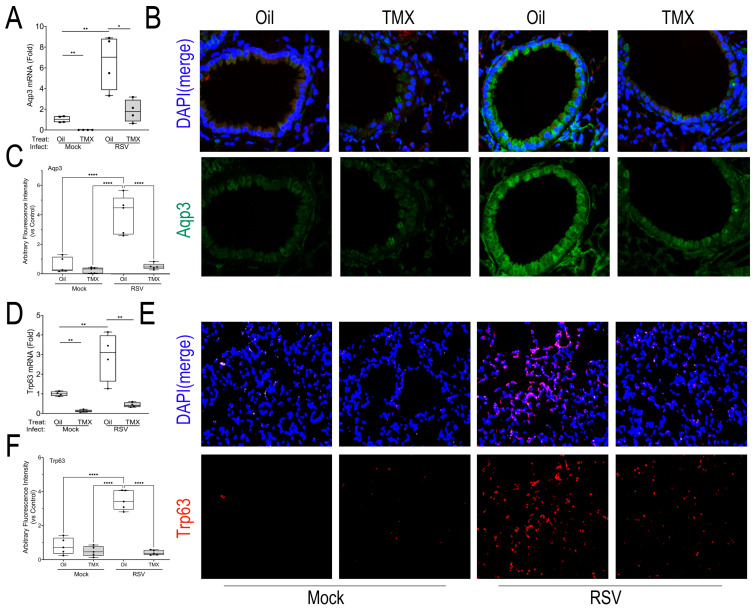
Epithelial atypia in RSV-PVLD. (**A**) Q-RT-PCR for *AQP3* mRNA in total lung RNA normalized to Gadph as internal control. A 25–75% IQR is shown; each symbol is an independent animal. * *p* < 0.05; ** *p* < 0.01. (**B**) IFM for AQP3 in a representative image from each treatment group. At the top, a merged image of AQP3 (green) fluorescence with DAPI nuclear stain (blue) is shown. At the bottom, AQP3 IFM only is shown. Scale bar, 40 mm. (**C**) The quantitation of total AQP3 IFM; AU, arbitrary fluorescence units. Each symbol is an average of multiple images for each individual animal (n = 5). **** *p* < 0.0001. (**D**) Q-RT-PCR for Gadph-normalized *TRP63* mRNA in total lung RNA. ** *p* < 0.001. (**E**) IFM for TRP63 in a representative image. At the top, a merged image of TRP63 (red) fluorescence merged with DAPI nuclear stain (blue) is shown; 40X. At the bottom, TRP63 IFM only is shown. Scale bar, 40 mm. (**F**) The quantitation of TRP63 in IFM in AU. **** *p* < 0.0001.

To better characterize the effects of RSV infection in epithelium, we stained for the presence of reparative progenitor cell markers. We first analyzed the expression of the small membrane transporter, aquaporin 3 (AQP3), a marker of acute lung injury associated with epithelial proliferation and migration [39]. In WT mice, we observed that RSV infection produced a significant 6.5 ± 2.6-fold increase in *AQP3* mRNA in lung (*p* < 0.01, n = 4–6; Figure 5A). This induction was significantly reduced to 1.8 ± 1.1-fold in the RSV-infected RelA CKOs (*p* < 0.01, n = 4–6; Figure 5A). To examine the tissue distribution of AQP3, immunofluorescence microscopy (IFM) was conducted, where increased AQP3 staining was observed in the membrane in small bronchioles in the basal cell layer and throughout the alveoli (Figure 5B). The quantitation of the IFM staining confirmed a 4.5-fold increase in the AQP3 immunofluorescent signal in RSV-infected WT mice that was normalized by RelA CKO (*p* < 0.01, n = 4–6; Figure 5C).

We next quantitated the tumor-related protein 63 (TRP63), a marker of alveolar progenitors responsible for repopulating the alveolus in response to bleomycin injury [40] and influenza H1N1 infection [20]. We found that RSV induced a 2.9 ± 1.2-fold increase in *TRP63* mRNA in RSV-infected WT mice (*p* < 0.01, n = 4–6), which was significantly reduced in both mock-infected and RSV-infected RelA CKO mice (*p* < 0.01, n = 4–6; Figure 5D). By IFM, TRP63 was not identified in mock- or RSV-infected bronchioles, consistent with other reports [41]. However, we did observe an increase in TRP63 immunostaining in a patchy distribution throughout the alveolar parenchyma in RSV-infected WT mice. This pattern was reduced in the RSV-infected RelA CKO (Figure 5E,F), indicating the TRP63 expression in *Scgb1a1*+ progenitors is downstream of RelA.

To further explore this finding, we examined the expression of ITG-α6 and β4 isoforms. We were particularly interested in this measurement because the expression of ITG is TRP63-dependent and would therefore serve as a marker of functional TRP63 activity [42,43]. We found that RSV induced *Itga6* mRNA by 3.7 ± 1.2-fold in RSV-infected WT mice (n = 4, *p* < 0.01, Supplementary Figure S2), an induction blocked by RelA CKO (Supplementary Figure S2). In striking contrast to that seen in the acute RSV infection, five-fold upregulation of *Itgb4* mRNA was observed (Supplementary Figure S2). Collectively, these data indicate that RelA signaling in *Scgb1a1*+ precursors is required for the expansion of the AQP3+/TRP63+/ITGα6+ epithelial cell progenitors in distal airways in RSV-PVLD.

### 2.6. Scgba1a1 Progenitors Contribute to the aAT2 Population in RSV-PVLD

**Figure 6:**
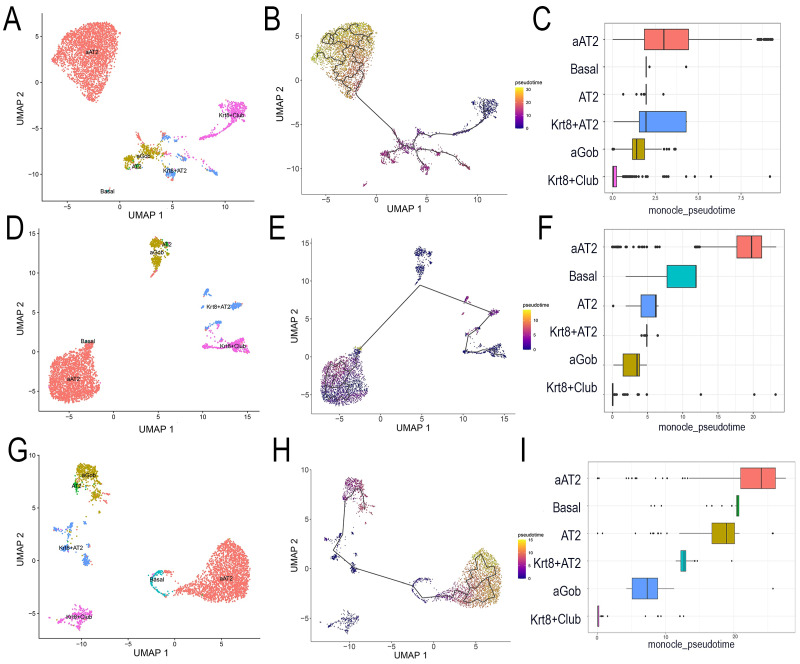
Epithelial cell trajectory analysis by pseudotime. Epithelial cells were subsetted from the scRNA-seq, resulting in distinct UMAP clustering. (**A**–**C**) represent the mock-infected mice; (**D**–**F**) represent the RSV-infected mice; (**G**–**I**) represent RelA CKO. (**A**,**D**,**G**) show relevant cell identities on the UMAP clustering. (**B**,**E**,**H**) are pseudotime trajectories. (**C**,**F**,**I**) show pseudotime box plots for each cell population. Color represents arbitrary pseudotime. Note that the *Scgb1a1+ Krt8+* club cell population appears as the root population in the WT samples, but it does not contribute to the differentiation trajectory in the RelA CKO (see Discussion).

We next conducted the trajectory/pseudotime analysis [44] to obtain a glimpse of the evolution of the *Scgb1a1*+ population. Here, epithelial cells were selected, reclustered, and subjected to pseudotime analysis. In mock-infected cells, a defined cell trajectory originating from the *Scgb1a1+ Krt8+* club cell population transitioning to Goblet cells (Gob) and terminating in the aAT2 population could be clearly identified (Figure 6B), with an increasing pseudotime gradient across this trajectory (Figure 6C). In the RSV-infected samples, the cell trajectory pathway does not substantially change, which again indicates that the *Scgb1a1+ Krt8+* club cell population serves as a root cell for the aAT2 terminal state. However, we noted that the rate of formation of the aAT2 population was significantly ~10-fold longer than in the mock-infected samples (Figure 6F). Interestingly, the Krt8+ club cells in the RelA CKO did not evolve into aAT2 population, suggesting that the RelA signaling is required for cell state transition (see Discussion).

**Figure 7:**
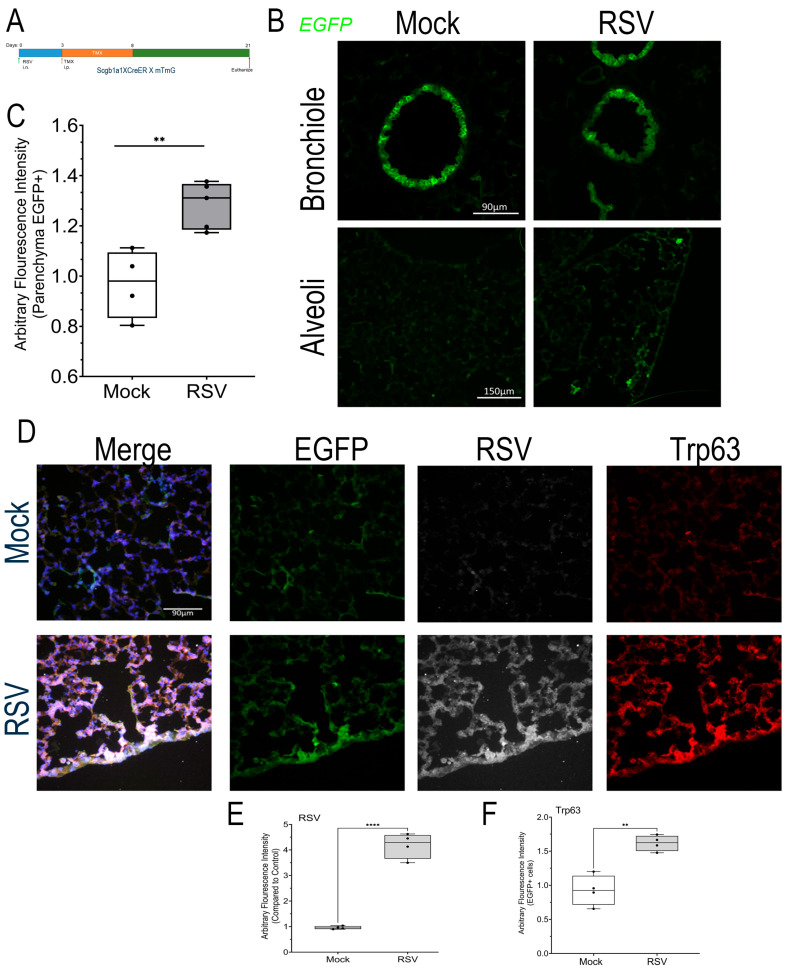
Lineage tracing of *Scgb1a+* progenitors. (**A**) A schematic view of lineage-tracing experiment. RSV-PVLD was first induced, followed by TMX treatment 3–8 d prior to harvest at d21 post-infection. (**B**) GFP fluorescence in bronchiole (**top**) and alveoli (**bottom**) for mock- or RSV-infected mice. A representative image from each treatment group is shown. (**C**) The quantitation of total GFP in alveoli. AU, arbitrary fluorescence units. Each symbol is an average of multiple images for each individual animal (n = 4). ** *p* < 0.01 *t* test. (**D**) IFM for EGFP (green), RSV F glycoprotein (white), and TRP63 (red) in mock-infected (top) or RSV-infected mice (bottom row). A representative image from each treatment group is shown. (**E**) The quantitation of RSV F glycoprotein staining. AU, arbitrary fluorescence units. Each symbol is average of multiple images for each individual animal (n = 4). **** *p* < 0.0001 post hoc. (**F**) The quantitation of TRP63 staining. ** *p* < 0.01 post hoc.

To directly establish the fate of the *Scgb1a1+* progenitor population in RSV-PVLD, we conducted cell lineage tracing studies by tagging *Scgb1a1*-expressing cells with GFP using heterozygous *Scgb1a1*-CreER^TM^ X dimer Tomato (mT) membrane-targeted green fluorescent protein (mG) mouse (mTmG) strain. These mice express WT levels of RelA and can be used to track the cellular lineage using the green fluorescent protein (GFP) reporter. All cells in the mT/mG mouse express Tomato, a red fluorescence protein, prior to Cre activation. In response to Cre activation, expression of Tomato is silenced and GFP is induced, enabling identification of *Scgb1a1*+ progenitors. Our previous light sheet microscopy studies using *Scgb1a1*-CreER^TM^ X mTmG mice mapped *Scgb1a1* progenitors as widely and uniformly distributed throughout the small bronchiolar epithelium [26]. We therefore subjected heterozygous *Scgb1a1* CreER^TM^ X mTmG mice to RSV-PVLD and labeled the *Scgb1a1*+ progenitor population after RSV infection (schematically shown in Figure 7A).

In either mock- or RSV-infected mice, GFP + *Scgb1a*-derived progenitors populated the small bronchioles, consistent with the role of these self-replicating cells as progenitors of secretory goblet and ciliated cells determined by others [45] and our cell trajectory analysis (Figure 7B). We next examined the population of GFP + *Scgb1a* in the alveoli. Here, we observed a low level of GFP+ cells consistent with presence of *Scgb1a1*-expressing cells in alveoli [45] (Figure 7B, bottom). We further noted that this population increased with RSV-PVLD. The quantification of the GFP+ cell population showed a RSV infection-induced GFP fluorescence from 0.96 ± 0.14 to 1.2 ± 0.09 arbitrary fluorescence units (n = 4, *p* < 0.01, Figure 7C).

To determine whether these progenitors contributed to the TRP63+ population, we conducted immunofluorescence microscopy (IFM) assays. Here, we found that the alveolar GFP+ population was positive for RSV F protein expression, where we observed a 4.18 ± 0.5-fold increase in the RSV F signal (n = 4, *p* < 0.001, Figure 7D,F). We also noted that the majority of GFP+ alveolar parenchymal populations co-stained with TRP63, where a 1.62 ± 0.11-fold increase in TRP63 staining was quantitated (n = 4, *p* < 0.001, Figure 7D,E). Collectively, these data indicate that *Scgb1a1+* progenitor cells are direct precursors of the atypical TRP63+ progenitors in the alveolar epithelium

### 2.7. RSV-PVLD Dysregulates Inflammatory Clock Genes in the Alveolar Mesenchymal Niche

**Figure 8:**
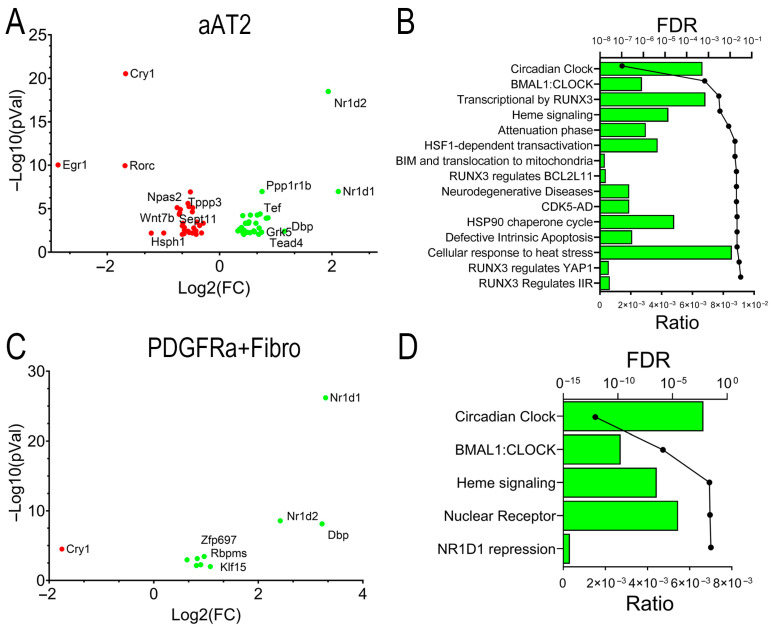
RSV-induced differentially expressed genes (DEGs). The pseudobulk analysis of DEGs in response to RSV infection. Differential gene expression in mock-infected, vehicle-treated aAT2 cells (**A**,**B**) or *Pdgfra*+ fibroblasts (**C**,**D**) was estimated with pseudobulk methods, correcting for multiple hypothesis testing [46]. (**A**,**C**) Volcano plots of Log_2_FC gene expression vs. −Log10(pVal) for significant genes are shown. Highly up- and downregulated genes are shown. (**A**) aAT2 cells; (**C**) *Pdgfra*+ fibroblasts. Selected abbreviations: Cry1, cryptochrome circadian regulator 1; Cry1, cryptochrome circadian regulator 1; Dbp, D-box binding PAR BZIP transcription factor; Egr1, early growth response 1; Nr1d, nuclear receptor subfamily 1 group D member; Rorc, RAR-related orphan receptor C. (**B**,**D**) Genome ontology enrichment of DEGs. Top 15 biological pathways identified for DEGs are shown. For each pathway, the fraction of genes represented in the pathway (ratio, bars) and the adjusted false discovery rate (FDR, circles) are shown. (**B**) aAT2 cells; (**D**) *Pdgfra* + fibroblasts.

Distal airway progenitors are engaged in a bidirectional trophic interaction with fibroblast populations in spatially resolved “niches”. In the distal parenchyma, *Scgb1a1+* progenitors are engaged with trophic interactions with PDGFRa+ fibroblasts and Hif1+ pericytes to maintain stem cell properties [21]. To further explore the effect of RSV infection in alveolar cell atypia, a differential gene expression (DEG) analysis of individual cell groups was conducted using pseudobulk analysis and DESEQ2 to minimize false positives [46]. We identified DEGs in aAT2 cells for mock-infected vs. RSV-infected WT mice. A total of 64 DEGs were significantly changed at an absolute value of log2FC > 1.5 and an adjusted *p*-value (pAdj) of <0.01. These genes were plotted in a Volcano plot representation, where nuclear receptor subfamily 1 group D member (*Nr1d*)-2 and -1 genes, also known as REV-ERB-β/α key regulators of inflammatory clock genes, were identified as being the most highly upregulated by RSV infection, with a reduction in cryptochrome circadian regulator 1 (*Cry1*) and early growth response 1 (*Egr1*) mRNAs (Figure 8A). A genome ontology analysis identified that the most highly affected biological pathways were that of circadian clock, RUNX3 signaling, heat shock factor (HSF1) transactivation, and others (Figure 8B).

A similar analysis was conducted for the *Pdgfr1a*+ fibroblasts. Here, RSV affected 66 genes at the same stringent cut-off values. Intriguingly, *Nr1d1* was the most highly induced, along with D-box binding PAR BZIP transcription factor (*Dbp*) and *Nr1d2*, along with a downregulation of *Cry1* (Figure 8C), mapping to a highly enriched circadian clock pathway (Figure 8D). Collectively, these data indicate that RSV-PVLD is associated with the dysregulation of inflammatory circadian clock genes in both aAT2 epithelial cells and *Pdgfr*a+ fibroblasts.

**Figure 9:**
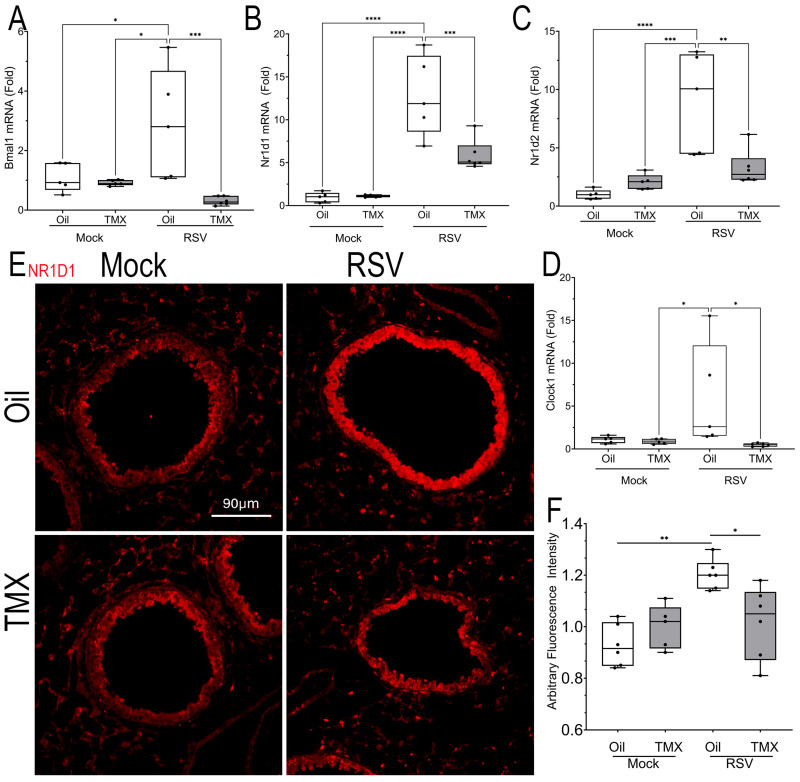
Aberrant expression of *BMAL-Nr1d1* clock genes. (**A**–**D**) Q-RT-PCR for BMAL-Nr1d1 pathway genes in total lung RNA normalized to Gadph as internal control. A 25–75% IQR is shown; each symbol is an independent animal. (**A**) *Bmal* mRNA, (**B**) *Nr1d1* mRNA, (**C**) *Nr1d2* mRNA, (**D**) Clock1 mRNA. * *p* < 0.05; ** *p* < 0.01, *** *p* < 0.001, **** *p* < 0.0001, post hoc. (**E**) IFM for NR1D1. A representative image from each treatment group is shown. Scale bar, 90 mm. (**F**) The quantitation of NR1D1 expression in AU. * *p* < 0.05; ** *p* < 0.01, post hoc.

The effect of RSV-PVLD on *Bmal* was validated using Q-RT-PCR. Here, we observed that RSV infection induced *Bmal* mRNA expression by 2.87 ± 1.9-fold in WT mice (n = 4, *p* < 0.05, Figure 9A), whereas this induction was reduced to 0.31 ± 0.44-fold in RSV-infected RelA CKO (n = 4, *p* < 0.05, Figure 9A). The *Nr1d1* mRNA expression was dramatically upregulated to 12.8 ± 0.44-fold in RSV-infected WT mice (n = 4, *p* < 0.0001, Figure 9B), and it was reduced in the RSV-infected RelA CKO mice to 5.8 ± 1.8-fold (n = 4, *p* < 0.001, Figure 9B). Similar patterns of RSV induction and inhibition by RelA CKO were observed for the 9.1 ± 3.2 -fold induction of *Nr1d2* mRNA and 5.9 ± 6.2-fold *clock* mRNAs (Figure 9C,D).

To confirm the upregulation of NR1D1, we conducted an IFM where a 1.6-fold increase in NR1D1 expression could be identified in RSV-infected WT mice, predominately in the bronchioles and to a lesser extent in the alveoli, an induction reduced by the RelA CKO (Figure 9E,F). Collectively, these data indicate that RSV-PVLD is associated with a dysregulated inflammatory clock gene expression.

### 2.8. RSV Disrupts Intercellular Epithelial–Mesenchymal Signaling Networks

Trophic mesenchymal–epithelial interactions mediate progenitor epithelial cell differentiation and myofibroblast expansion in response to injury through secretion of matricellular and soluble growth factors [21]. Our collective findings, that RSV-PVLD induces changes in epithelial–mesenchymal populations and their phenotypes, suggested to us that RSV infection may affect these trophic signaling networks. To better understand this, we conducted a systematic inference of epithelial–mesenchymal cell communication networks. Here, scRNA-seq data was analyzed using a validated, probabilistic method that incorporates the expression of secreted factors, as well as their receptors and co-receptors, across a manually curated database of >2000 interactions [47].

**Figure 10:**
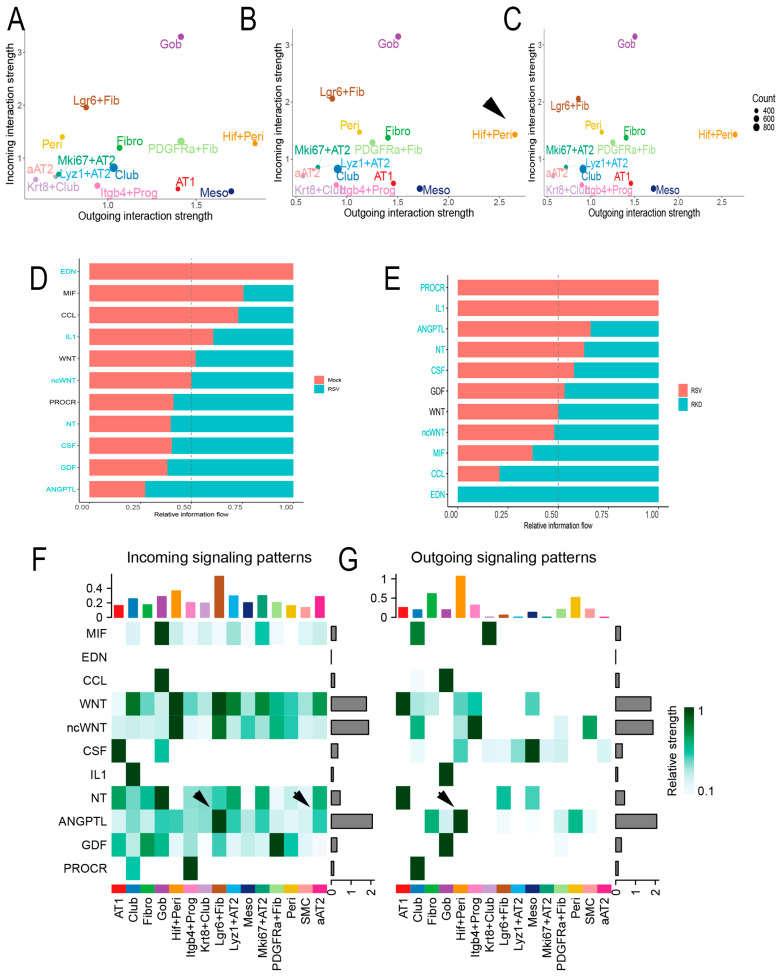
Intercellular signaling analysis of epithelial–mesenchymal niches. (**A**–**C**), 2D global cellular communication network analysis. Each dot represents the communication network of one cell group with incoming signal plotted on the *x*-axis and outgoing signaling plotted on the *y*-axis. Dot size is proportional to the overall communication probability. (**A**), Mock-infected WT; (**B**), RSV-infected WT; (**C**) RSV-infected RelA CKO mice. Note the increase in outgoing signals generated by the *Hif1*+ Peri (arrowhead). (**D**), Information flow in RSV vs. mock-infected mice. All the significant signaling pathways identified were ranked based on their differences of overall information flow between mock and RSV infected mice. The overall information flow of a signaling network is calculated by summarizing all the communication probabilities in that network. The top signaling pathways colored by red are more enriched in mock-infected mice, and the bottom ones in cyan are enriched in RSV-infected mice. Signaling pathways are shown on the *y*-axis; those colored in red or cyan are significant between treatments at *p* < 0.05 level. (**E**), Information flow in RSV infected vehicle mice and RSV-infected RelA CKO (RKD). (**F**) Heat map of incoming signaling of individual cellular communication pathways for cell types in mock-infected mice. The top colored bar plot shows the total signaling strength of a cell group by summarizing all signaling pathways displayed in the heatmap. The right gray bar plot shows the total signaling strength of a signaling pathway by summarizing all cell groups displayed in the heatmap. Note the increase in ANGPTL4 incoming signal in *Lgr6*+ Fib and aAT2 (arrowheads). (**G**), Heat map of outgoing signaling for cells in RSV-infected WT mice. Note the increase in ANGPTL4 by *Hif*+ Peri (arrowhead).

We individually analyzed each treatment condition for dominant “senders” (those cells producing factors) and “receivers” (those cells expressing cognate receptors and co-factors). These data were plotted into a two-dimensional representation of outgoing signals vs. incoming signals (Figure 10). In mock-infected WT mice, the dominant cell types producing outgoing signals included Goblet cells (Gob) and *Lgr6*+ alveolar fibroblasts (*Lgr6*+ Fib, Figure 10A). We paid particular attention to this effect in the *Lgr6*+ Fib population, as epithelial trophic signaling is important for its known role supporting small airway epithelial progenitors [21]. By contrast, in RSV-infected WT mice, *Hif1*+ Peri cells were highly induced to produce outgoing messages (arrowhead, Figure 10B). In the RelA CKO, the outgoing signals of Hif1+ Peri population were unchanged relative to RSV-infected WT mice (Figure 10C).

The intercellular signaling intensity for specific pathways was determined by condition and cell type and displayed as an information flow bar chart (Figure 10D). In mock-infected WT mice, the endothelin (EDN), macrophage migration inhibitory factor (MIF) and CC chemokine ligand (CCL) pathways were the highest fraction observed (Figure 10D). By contrast, CSF, growth differentiation factor (GDF), and angiopoietin-like (ANGPTL) pathways emerged as the top enriched pathways in RSV-infected vehicle-treated mice (Figure 10D). Of these, ANGPTL4 signaling was noted because mesenchymal ANGPTL signaling controls intestinal stem cell regeneration and fibroblast expansion in response to lung [48] and intestinal injury [49]. By contrast, a distinct pattern of information flow was observed in the RSV-infected WT vs. RelA CKO. Here, protein kinase c receptor (PROC), IL1, and ANGPTL signaling emerged as the top pathways in RelA CKO, along with ANGPTL4 (Figure 10E).

To better understand the intercellular signals converting on the aAT2 population, we analyzed the incoming signals as a function of cell type for mock and RSV infected WT mice. These data are displayed as a heat map, where a significant enhancement of ANGPTL signaling was seen in the RSV-infected cells as an incoming signal for the *Lgr6*+ fibroblasts and aAT2 cells (arrowheads, Figure 10F). The majority of cell types producing ANGPTL4 were *Hif*1+ Peri and Fibro (arrowheads, Figure 10G). Collectively, we interpreted these data as indicating that RSV-PVLD is associated with dysregulated mesenchymal ANGPTL4 signaling from *Hif1*+ Peri to *Lgr6*+ alveolar fibroblasts and the aAT2 cell population.

### 2.9. RSV Dysregulates Mesenchymal–Epithelial ANGPTL Signaling

**Figure 11:**
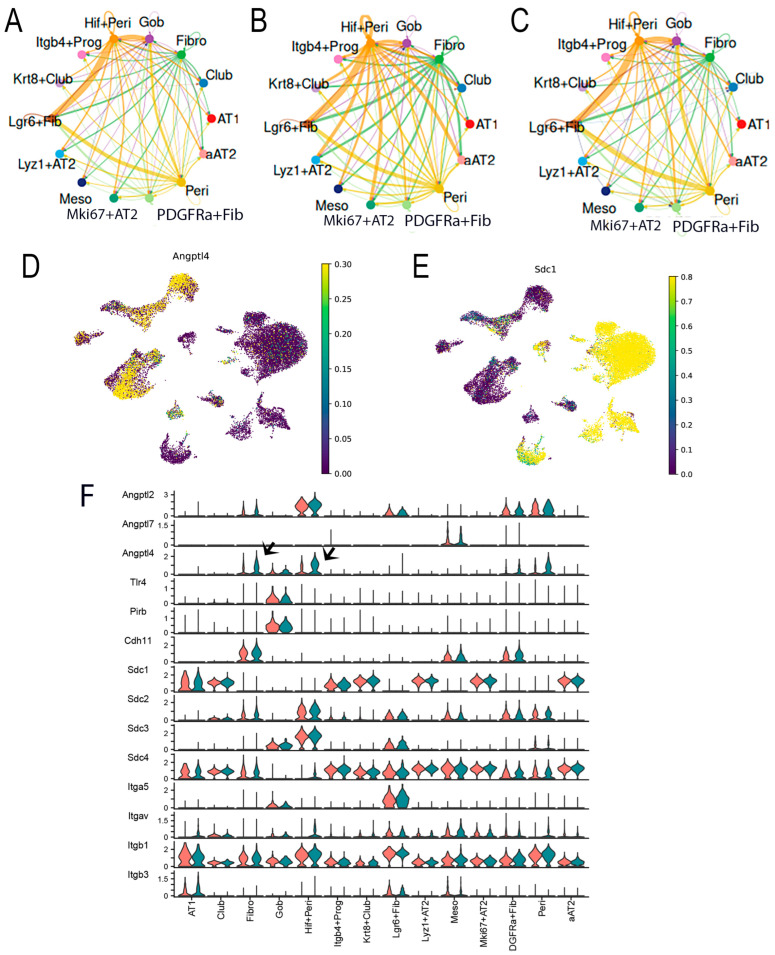
RSV dysregulates a mesenchymal–epithelial ANGPTL4 signaling pathway. (**A**) Circle plot of ANGPTL4 communication probability between cell types. Each connecting edge width represents the communication probability. Note the autocrine regulation of ANGPTL4 on the *Hif1* + Peri and *Lgr6* + Fib and increased communication probability with Gob cells. (**B**), Circle plot of ANGPTL4 communication for RSV infected WT mice. Note increased signaling by *Hif +* Peri and fibroblasts. (**C**) Circle plot of ANGPTL4 for RSV-infected RelA CKO. (**D**) *ANGPTL4* expression in cells from RSV-infected mice. UMAP representation colored by *ANGPTL4* mRNA expression. (**E**) Expression of major ANGPTL4 receptor. *Sdc1* expression overlaying UMAP representation for RSV-infected cells. Note the high ANGPTL4 receptor expression in aAT2 and *Krt8*+ club and club cells. (**F**) Systematic analysis of ANGPTL family ligands and known receptors. Violin plot showing the expression distribution of signaling genes and receptors for the ANGPTL4 pathway. For each plot, expression is shown for mock (orange) or RSV-infected mice (green). Note the upregulation of ANGPTL4 ligand expression in *Hif*+ Peri and Fibro (arrowheads) in RSV-infected mice without changes in ANGPTL receptor expression in target epithelial cells.

To better understand the intercellular signaling network of ANGPTL in RSV-PVLD, we analyzed the strength of ANGPTL signaling between individual cells as a function of treatment. In mock-infected mice, autocrine ANGPTL is produced by *Hif1* + Peri cells that signal to both *Lgr6*+ Fib and Goblet cells (Figure 11A). By contrast, ANGPTL signaling intensity is increased in RSV-infected mice, where *Hif1*+ Peri signaling to aAT2 cells becomes prominent, and ANGPTL signaling increases in Fibro to aAT2, *Mki67* + AT2, *Lyz* + AT2, and *Lgr6*+ fibroblasts (Figure 11B). In RelA CKO, the ANGPTL patterns largely return to those of mock-infected controls (Figure 11C). In airway epithelial cells, syndecan-1 (Sdc1) is the dominant ANGPTL4 receptor. Overlaying *ANGPTL4* and *Scd1* expression patterns in UMAP, we observed that *Hif1*+ Peri and *Pdgfra*+ Fibro cells express *ANGPTL4* (Figure 11D), whereas aAT2 and *Krt8 + Scgb1a*+ club cells have the highest *Scd1* expression (Figure 11E), further suggesting the presence of mesenchymal–epithelial ANGPTL signaling.

To systematically identify the ligands and receptors in the ANGPTL pathway, the levels of expression for each in RSV-infected vehicle-treated vs. control mice were quantified and plotted as violin plots. Although *Angptl2* mRNA was constitutively expressed in mesenchymal cells, *ANGPTL4* mRNA was upregulated in RSV-infected Fibro and *Hif* + Peri (arrowheads, Figure 11F).

**Figure 12:**
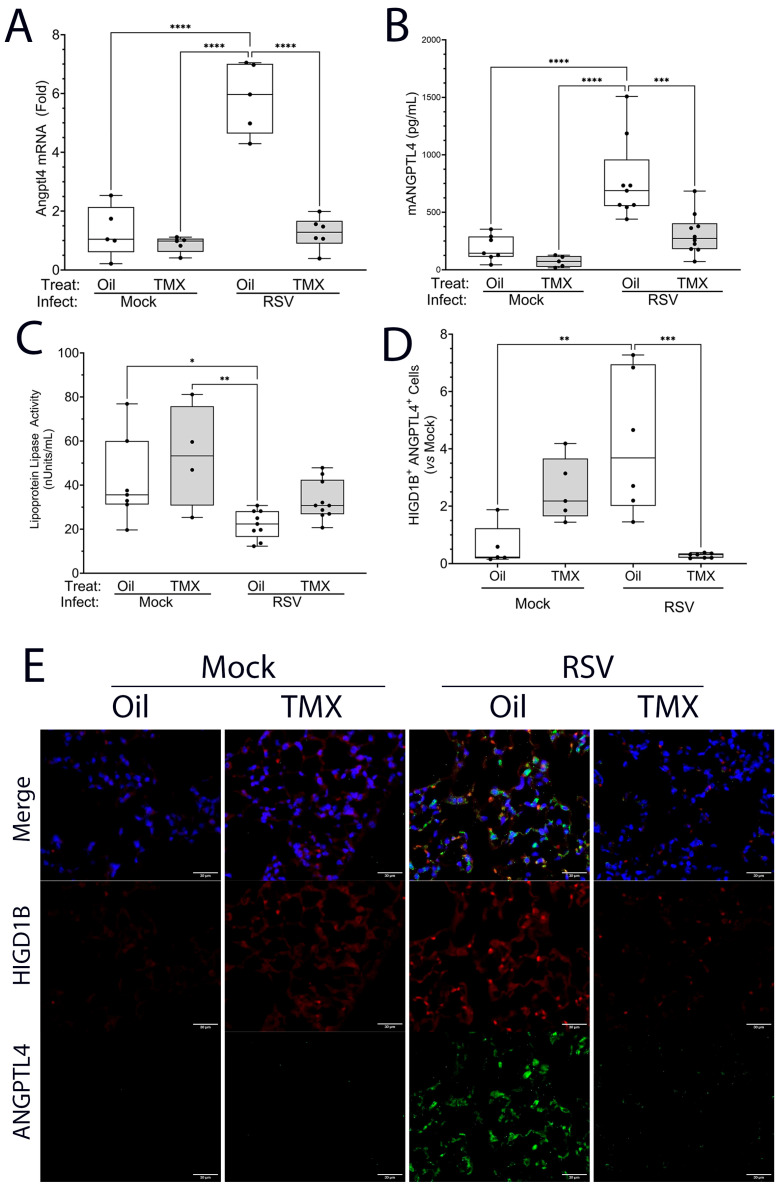
Upregulation of biologically active ANGPTL4 in mesenchymal niche. (**A**) Changes in ANGPTL4 mRNA abundance (n = 4). ANGPTL4 mRNA was analyzed by Q-RT-PCR normalized to Gadph as internal control. A 25–75% IQR is shown; each symbol is an independent animal. **** *p* < 0.0001. (**B**) ANGPTL4 abundance. mANGPTL4 protein concentration in BALF was determined by ELISA (n = 4). BALF concentration values are shown; each symbol is an independent animal. *** *p* < 0.001; **** *p* < 0.0001. (**C**) Lipoprotein lipase inhibitory activity. BALF extracts from indicated treatments were incubated with LPL and enzymatic activity determined. * *p* < 0.05; ** *p* < 0.01 post hoc. (**D**) The quantitation of ANGPTL4-expressing pericytes. Immunofluorescence staining for HIGD1B and ANGPTL4 was conducted. The quantitation of ANGPTL4+ pericytes from multiple independent fields is shown. ** *p* < 0.01, *** *p* < 0.001 post hoc. (**E**), Immunofluorescence staining for HIGD1B (Red) and ANGPTL4 (green) from a representative field of view. Image is at 40X magnification, scale bar is 30 μm.

To confirm the upregulation of the ANGPTL4 pathway, *ANGPTL4* mRNA abundance was independently determined in RSV-PVLD by Q-RT-PCR. We observed that RSV induced a 5.9 ± 1.2-fold increase in total lung *ANGPTL4* mRNA that was reduced to 1.3 ± 0.55-fold in RelA CKO mice (n = 4, *p* < 0.0001, Figure 12A). To determine if the protein was secreted into the BALF, the ANGPTL4 concentration was measured through ELISA; we observed that the ANGPTL4 concentration of 217 ± 123 pg/mL in mock-infected mice rose 4.2-fold, to 920 ± 424 pg/mL in RSV-infected mice (*p* < 0.001, n = 5; Figure 12B); this induction was reduced by ~50% to 404 ± 163 pg/mL in RelA CKO mice (*p* < 0.01, n = 5; Figure 12B) in a manner paralleling the changes in mRNA expression. Collectively, these data confirm the Cell Chat predictions that RSV activates ANGPTL4 signaling at the level of ligand expression.

We next sought to determine if we could detect ANGPTL4 biological activity. For this purpose, we leveraged previous work showing that ANGPTL4 is a reversible, noncompetitive inhibitor of lipoprotein lipase (LPL) activity [50]. BALF extracts were added to a mixture of LPL and its quenched fluorogenic triglyceride substrate, whose hydrolysis released quantitative fluorescence. Relative to mock-infected mice, we observed that the BALF from the RSV-infected WT mice had significant LPL-inhibitory activity, whereas the BALF from RelA CKO had undetectable inhibitory activity (Figure 12C).

Finally, to determine whether *ANGPTL4* was being produced by pericyte niche, we quantitated the pericyte population using the hypoxia-inducible domain family member 1B (HIGD1B), a selective lung pericyte marker [51]. Here, we found that RSV-PVLD induced a significant 4.2 ± 2.5-fold upregulation of ANGPTL4+/HIGD1B+ cells (*p* < 0.0001, n = 4), indicating the expansion of the pericyte population (the quantitation of the double-staining population is shown in Figure 12D; representative images are shown in Figure 12E). Collectively, we conclude that RSV-PVLD upregulates the ANGPTL4 expression in the pericyte population and that this upregulation is dependent on NFκB signaling in the *Scgb1a1+* progenitor population.

## 3. Discussion

Children with RSV LRTIs are at greater risk for long-term reductions in lung function and are at a two-fold increased risk of premature death from a respiratory disease [3,4,5,6]. As the target permissive for RSV infection, the epithelium plays a primary role in modulating an acute RSV disease through an inducible innate response [15,16,17]. However, the role of innate signaling in long-term remodeling outcomes of a RSV disease is not fully understood. Here, we utilize a well-established inducible knockout in a key sentinel epithelial cell to understand the mechanistic role of RelA in the determination of long-term sequelae of early-life RSV infection. We find that the RelA signaling in *Scgb1a1*+ progenitors mediates major mucosal changes in RSV-PVLD by promoting the expansion of atypical, progenitor-like alveolar epithelial cells with dysregulated circadian clock expression. In addition to identifying the presence of latent RSV in aAT2, we find evidence that RSV modulates epithelial–mesenchymal intercellular ANGPTL4 signaling pathways. Schematically illustrated in Graphical Abstract, we conclude that PVLD is the end-product of a spatiotemporally coordinated, multicellular reparative response to lower airway viral infection mediated by dysregulated epithelial–mesenchymal trophic signals.

### 3.1. Early-Life RSV Infections Induce Epithelial Atypia Through Epithelial Plasticity

In rodent models, RSV infections in early life produce chronic changes that mimic LRTIs in humans [32,33]. For example, it has been reported that RSV infections in neonatal mice produce long-term inflammatory airway disease characterized by airway functional changes (obstruction and hyper-reactivity [52]), as well as histological pathology (peribronchial inflammation, bronchial wall thickening, and subepithelial fibrosis [53]). Recent work on early life infections in the BALB/C model have described the emergence of an indeterminant AT1-AT2 population associated with persistent IL33 expression [54]. This study found that early-life exposure to IL1b triggers a similar pattern of IL33 expression, suggesting that innate responses initiate some of the chronic changes in RSV-PVLD.

The BALB/C model is widely used to understand the linkage between RSV and allergy because this strain exhibits a robust Th2 response, potentially obscuring the contributions of epithelial intrinsic immunity on airway modeling. Consequently, we studied RSV-PVLD in the C57BL6/J strain, a strain that mounts a robust innate and Th1- polarized immune response [55]. Here, we observe that an acute RSV infection produces loss of distal ciliated epithelial cells and confirm the rapid emergence of atypical aAT2 cells using colocalization in IFM. These alveolar epithelial changes evolve over time in RSV-PVLD, driven by IL33 and associated with later upregulation of ITG isoforms and dysregulation of the circadian clock genes (Graphical Abstract). Our work therefore extends these previous findings, providing direct evidence that innate signaling in Scgb1a1 progenitors is required for the full manifestation of RSV-PVLD.

### 3.2. Scgb1a1+ Progenitors Mediate Epithelial Atypia in RSV-PVLD

Understanding the mechanisms that control epithelial remodeling may shed light on the pathological sequelae of RSV LTRI. The presence of atypical AT2 cells may be due to dysregulated differentiation programs, as well as the emergence of progenitor populations. scRNA sequencing of pediatric nasal epithelial cells has shown that RSV infection in the first year of life is associated with atypical differentiation programs [56], a finding observed in human epithelial cells in ALI cultures [10]. In parallel, RSV induces ciliated cell loss and sloughing [57], which may serve as a trigger for the expansion of basal cell progenitors. Depending on the location and severity of injury, distinct populations of basal cells are recruited for airway repair.

Studies on the influenza (H1N1) and Sendai virus (SeV) infection models where extensive viral infection induces cellular necrosis, a basal cell regenerative response is activated to repopulate the lower airway [33,41,58,59]. Of particular interest to this study, this population is derived, in part, from *Scgb1a1*-expressing club cell progenitors originating in the broncho-alveolar junction [20,41,60]. Additionally, other lineage-tracing experiments in H1N1 infection found that these *Scgb1a1* progenitors become TRP63+ and contribute to AT2 repopulation [61]. The role of innate signaling in this population was not examined. These studies reinforce our findings that the TRP63+ *Scgb1a1*+ progenitor is an important determinant of injury-induced repair and resolution in the lower airway after RSV infection.

Our findings from the *Scgb1a1*-mTmG lineage-tracing experiments provide further, direct evidence that *Scgb1a1*+ progenitors contribute, at least in part, to the TRP63+/KRT8+ aAT2 population in RSV-PVLD. The phenotype of the aAT2 cells in this study are similar to that of the well-characterized “atypical differentiation-intermediate” (ADI), “damage-associated transitional” (DATP), or “pre-alveolar type-1 transitional cell state” (PATS) cells [20,62,63,64] identified in bleomycin and influenza injury. Single-cell analyses of this ADI population show that these cell types express characteristic epithelial–mesenchymal plasticity (EMP), cellular senescence, and TRP63+ signaling pathways [20]. Importantly, the persistence of this reparative cell population(s) is (are) associated with dysplastic repair and fibrotic remodeling. In bleomycin and viral injury models, for example, ADI cells contribute ~50% to the regenerating AT2 population, where their persistence results in dysplastic, fibrotic repair [20].

By contrast, in RSV-PVLD, *Scgb1a1+* progenitor population in RSV-PVLD appears to induce epithelial hyperplasia, resulting in defective ambient O_2_ exchange but not extensive interstitial fibrosis. More work will be required to establish the relationship of RSV-induced aAT2 cells with those of the ADIs arising in bleomycin injury. Although we can confirm the expression of KRT8 and TRP63, we can also identify an inflammatory clock signature in aAT2 cells, which is distinct from that reported in the ADI population. We have not determined the presence of cellular senescence, which will be further explored in future studies. Nevertheless, these data suggest to us that progenitor transitions can evolve into multiple distinct cell states, depending on the type of injury, consistent with our cell trajectory analysis.

### 3.3. RSV Modulates Epithelial Plasticity and Differentiation Programs Through Innate Signaling

Although the *Scgb1a1* lineage of TRP63+ progenitors have been implicated in alveolar repair, the mechanisms of how this population expands in the setting of RSV infection is not yet understood [65,66]. In other models of airway injury, regenerative epithelial changes are mediated through a growth factor-initiated epigenetic reprogramming leading to EMP [67,68]. Mucosal injury induces several important cell stress pathways important in EMP, including the unfolded protein response [69,70] and NFκB. Our previous work has placed RelA as a major mediator of the TGF- and viral-induced cellular plasticity in the lower airway basal epithelial cells [67,71]. These previous mechanistic studies found that RSV replication triggers EMP, a series of cell state transitions mediated by IKK-NFκB signaling. In TRP63+/KRT5+ models of basal cells in vitro, RelA signaling is a component of a transcription factor cascade that maintains paracrine TGFβ and Wnt signaling, sustaining EMP [21,70,71]. Plasticity programs play a central role in epithelial repair by disrupting the intercellular hemidesmosomal contacts with the basement membrane, enhancing the contractile protein expression that enhance cytokinesis, enabling basal cells to migrate and repopulate the areas of epithelial injury [70,72,73]. A crucial determinant of resolution is whether the EMP-transitioned cells fully differentiate into mature AT cells or become trapped in a differentiation-intermediate state, such as aAT2 or the ADI cells, where their persistence produces defective oxygenation or fibrosis [20].

An additional finding of our study is that the differentiation-indeterminant aAT2 cells have evidence of low levels of RSV F glycoprotein expression, suggesting that RSV latency may also be directly influencing the aAT2 cell phenotype. Of relevance, it has been reported that RSV replication alters differentiation programs of lower airway-derived TP63+ basal cells in ALI, redirecting them from a default differentiation program to ciliated cells into a program-generating mucous-secreting goblet cells [10]. This work further showed that inducible type I/III IFNs secreted from RSV-infected cells mediate reprogramming. Since we have shown that RSV-induced IFN synthesis is well-established to be dependent on RelA [74], collectively, these data suggest that both local growth factor-induced mesenchymal plasticity and direct viral replication-induced cellular reprogramming is centrally mediated by RelA.

### 3.4. RSV Antigen Persistence in RSV-PVLD

The evolution of acute RSV lower respiratory tract infection has been well-established in the mouse model [75]. Here, RSV replication in type 1 and type 2 alveolar epithelial cells can be seen [76], mimicking patterns of RSV replication in fatal human infections [9]. These findings are replicated in our strand-specific assay, where evidence of active replication is seen only in acutely infected mice. By 4–6 days after acute infection, the virus is largely cleared from the BALF. Interestingly, we detect persistent RSV transcription and cellular expression of the RSV F glycoprotein but are unable to detect infectious virions in the BALF or active replication intermediates using a more sensitive RT-PCR assay. These data are consistent with the findings of Schwartze et al., who reported low level of RSV transcription and protein expression in wild type Balb/c mice after the RSV infection [77]. Similar findings were described by Bannister et al., who described a phenomenon of “abortive” RSV replication in a mouse model [35]. This level of RSV replication was not sufficient to be detected in the plaque assay of BALF.

### 3.5. RSV Disrupts Circadian Clock Gene Expression in aAT2 Cells

The current study is the first to describe coordinate *Nr1d-1/-2* mRNA induction in aAT2 epithelial cells, as well as *Pdgfra* + fibroblasts in RSV-PVLD. Our experiments were designed to control for variations in clock gene expression, where the timing of infections and harvest were synchronized to the same time. We think that this finding is important because the coordinated upregulation of *Nr1d-1* and *-2* with downregulation of *Cry1* mRNA suggests to us that RSV-PVLD is associated with a dysregulated circadian clock function in epithelial–mesenchymal niche cells. *Nr1d* is an inflammation-associated nuclear transcription factor controlling lipid metabolism, cytokine activation, and the activation of *Bmal1* in an oscillatory transcription termination loop [78].

Clock genes are cyclically expressed in the lung parenchyma, whose expression levels determine fibrosis, inflammation, and immunity [79,80]. In mice, *Bmal* deficiency enhances replication, remodeling, and disease severity in response to SeV replication [81], linking the BMAL pathway as a determinant of anti-viral response. Moreover, we note that bronchial hyperreactivity chronically persists after the RSV infection in mice [52] and that a dysregulation of BMAL has been linked to bronchial hyperreactivity in human asthma [81]. More work will need to be done to investigate the functional significance of this intriguing finding of RSV-induced dysregulation of the clock.

### 3.6. RSV-PVLD Disrupts Epithelial–Mesenchymal Niches

Basal epithelial cells are maintained in the stem cell through trophic interactions with mesenchyme through paracrine signals that modulate their differentiation and proliferation [21]. To understand these interactions affected by RSV-PVLD, we systematically identified cell–cell interactions using a probabilistic (“Cell Chat”) analysis of our scRNA-seq data. Our findings show that the *Scgb1a1* + and aAT2 epithelial cells are actively engaged with trophic and inflammatory intercellular signaling pathways with *Pdgfra*+ fibroblasts and Hif1+ pericytes. We are aware of other spatial transcriptomics work that have identified *Scgb1a1+*-derived TRP63+ progenitor cells engaged in PDGFRa+ fibroblasts within a small airways “niche” [21,41,60,82]. Our findings suggest that multiple constitutive and RSV-inducible mesenchymal–epithelial signaling pathways include those of PROCR, IL1, and ANGPTL [21]. In this study, we demonstrate that ANGPTL4 is upregulated at the mRNA level by RSV-PVLD, associated with the presence of LPL inhibitory activity in the BALF. By immunostaining, a dramatic upregulation of ANGPTL4 is seen throughout the alveolar parenchyma associated with HIGD1B+ pericytes. Interestingly, RelA CKO in *Scgb1a1* + progenitors affect fibroblast and pericyte populations and signaling pathways, indicating complex, bidirectional signaling mediated by epithelial RelA with multiple mesenchymal populations [21].

### 3.7. A Potential Role for Angiopoietins in Post-Viral Lung Disease

Here, we validate the Cell Chat prediction for the upregulation of ANGPTL4 in RSV-PVLD. ANGPTL4 was of interest because this member of the angiopoietin family is a secreted factor important in metabolic transition, stem cell renewal, and fibrosis [83,84,85]. Moreover, this protein works as a chemotactic factor for ITGβ1/5-expressing cells in fibrosis [84] and functions as a regulator of Wnt signaling through the degradation of the LRP6 receptor [85]. Lastly, fibroblast-generated ANGPTL4 mediates bleomycin-induced fibrosis [48]. These intriguing findings provide plausible mechanism for role of ANGPTL4 in regulating Wnt-dependent differentiation in RSV-PVLD. In future studies, we will extend these findings to better understand the contribution of epithelial–mesenchymal niche in the anti-viral response.

## 4. Materials and Methods

### 4.1. Acute RSV Infection

The experiments were conducted using a protocol approved by the UW Institutional Animal Care and Use Committee (IACUC), with a 12 h light and 12 h dark cycle. Seven- to eight-week-old Scgb1a1-CreER^TM^ X RelA^fl/fl^ mice in the C57BL6/J background were mock infected (PBS) or infected with RSV (intranasally, 10^7^ PFU; mice of both sexes were used). *Scgb1a1*-CreER^TM^ X RelA^fl/fl^ mice in the absence of treatment were fully RelA wild type. The mice were euthanized after 4 days, wherein the tissue and BALF were collected (n = 4).

### 4.2. Plaque Assay

The HEP-2 cells were cultured in standard growth media (MEM, 10% FBS and 1%P/S) until confluent. The BALF collected from the lung was added using a ten-fold serial dilution. The cells were incubated at 37 °C for 1 h, and then a methylcellulose semi-solid overlay was added before incubating for 5 days at 37 °C, 5% CO_2_. On day 5, the overlay was removed, and the cells were fixed with 10% formaldehyde for 30 min. After the formaldehyde was removed, the cells were stained with a solution of 1% crystal violet in 70% ethanol for 30 min. The crystal violet was washed in water before imaging.

### 4.3. RSV-PVLD

The experiments were conducted using a protocol approved by the UW Institutional Animal Care and Use Committee (IACUC), with a 12 h light and 12 h dark cycle. The time of the RSV infection and Tamoxifen were at ZT8, while euthanasia was at ZT4. The *Scgb1a1*-CreER^TM^ X RelA^fl/fl^ mice (homozygous) were deficient in RelA with tamoxifen (TMX) administration. Cell lineage-tracing was conducted using *Scgb1a1* CreER^TM^ X mTmG mice, which exhibit a wild-type RelA response, along with Scgb1a1 progenitor tracing with GFP (Mock n = 4; RSV n = 5). In both the *Scgb1a1*-Cre ER^TM^ X RelA^fl/fl^ and *Scgb1a1* CreER^TM^ X mTmG mice, at day 0, the animals were mock- (PBS; n = 5) or RSV-infected 3–5 weeks after birth (intranasally, 10^7^ PFU; n = 6). Three days post-infection, the mice were randomized into the corn oil (10% ethanol and 90% corn oil; oil) or tamoxifen (TMX) treatment (20 mg/kg/d i.p.; Sigma Aldrich, St. Louis, MO, USA; T5648; RelA CKO) for 10 doses. As for the *Scgb1a1* CreER^TM^ X mTmG mice, all animals received TMX. The animals were then rested for an additional week (21d post-RSV infection) before oxygen saturations (MouseOx^®^ Plus, Starr Life Sciences Corp., Oakmont, PA, USA) were measured, and then the mice were euthanized, and the tissue and BALF were collected (n = 4–6).

### 4.4. Quantitative Real-Time PCR (Q-RT-PCR)

Total RNA from the left lung was extracted and cDNA was prepared using LunaScript RT SuperMix Kit (New England Biolabs, Ipswich, MA, USA) as previously described [25]. Gene specific primers used are shown in Supplementary Table S1. Relative changes in gene expression were quantified relative to control *Gapdh* transcripts using the ΔΔCt method.

### 4.5. Strand-Specific Reverse Transcriptase (RT)-Quantitative (q)PCR of RSV Replication

RSV replication was detected by strand-specific RT-qPCR, following the established methodology [35]. Firstly, strand cDNA was synthesized using the LunaScript Primer-Free master mix (E3025, New England Biolabs), with gene-specific primers targeted to the positive or negative sense RSV A2 nucleocapsid region RNA (Supplementary Table S1). Strand-specific detection through qPCR was performed using the Luna^®^ Universal qPCR Master Mix (New England Biolabs), as described above. The positive-sense RNA-specific primer and qPCR tag were used as the forward and reverse primer pair to quantify the positive nucleocapsid mRNA and RI RNA while the negative sense RNA-specific primer and qPCR tag were used as the forward and reverse primer pair to quantify the genomic RNA (Supplementary Table S1). Relative changes in gene expression were quantified relative to control *Gapdh* transcripts using the ΔΔCt method.

### 4.6. Immunohistochemistry

Right mouse lungs were frozen fixed in OCT (optimal cutting temperature, Tissue-Tek^®^ Sakura, VWR, Torrence, CA, USA). Using 95% ethanol and 100% acetone, the tissue was fixed to the slide before blocking with goat serum (1:1000; Invitrogen, Carlsbad, CA, USA). The sections were incubated with a primary antibody (Supplementary Table S2) overnight, washed, and stained with the appropriate secondary for 1 h. The slides were mounted using ProLong™ Gold Antifade containing DAPI (ThermoFisher Scientific; P36935, Waltham, MA, USA). The images were acquired on an ECHO Revolve R4 Microscope (ECHO, San Diego, CA, USA). Hematoxylin–eosin stain (H&E) was performed by the university TRIP laboratory (Translational Research Initiatives in Pathology) core services.

### 4.7. Single-Cell RNA Sequencing (scRNA-seq)

scRNA-seq libraries were constructed according to the Chromium NextGEM Fixed RNA Profiling Reagent Kits user guide (10X Genomics). The cells frozen in 10X Genomics Quenching Buffer were thawed and resuspended in 0.5X PBS with 0.02% BSA. The total cell number was quantified on the Luna-FX7 automated cell counter (Logos Biosystems, Anyang, Republic of Korea) using acridine orange/propidium iodide stain. Aliquots of less than two million cells were used as input for the probe hybridization reaction and incubated at 42 °C overnight. Following the incubation, subsets of four samples with unique probe barcodes were pooled and washed in 10X Genomics Post-Hyb Wash Buffer. The samples were filtered through 30 μm Celltrics filters (Sysmex, Kobe, Japan), and the appropriate volume of cells was loaded onto a Single Cell Chip Q. The GEMs were transferred to emulsion safe strip tubes for the cell and UMI barcoding reaction using an Eppendorf MasterCycler Pro thermocycler (Eppendorf, Hamburg, Germany). After the GEM incubation, the barcoded probe products were amplified through PCR. A portion of the amplified probe products was put into a second PCR reaction to add unique dual indexes to each library. The final libraries were quantified on the Qubit v4 fluorometer (ThermoFisher Scientific) and profiled on the Agilent 4200 Tapestation (Agilent Technologies, Santa Clara, CA, USA). These libraries were sequenced on a NovaSeq X+ sequencer with paired-end, 150 bp sequencing (Illumina, Inc. San Diego, CA, USA). The data was processed with bcl2fastq.

### 4.8. scRNA-seq Analysis

STAR (version 2.5.2a) was used for mapping. The reads were aligned to the mm10 reference genome, excluding barcodes with <200 detected genes. The numbers of cells in each library are shown in Supplementary Table S3. QA/QC processing showed that >90–93% of the sequencing reads were within cells, indicating negligible ambient RNA contamination (Supplementary Table S4). The cells with >10% mitochondrial reads and >5000 UMIs were filtered out from further analysis. The downstream analysis was performed in ScanPy (v. 1.12, Python 3.10). Doublets were identified and removed using the nearest-neighbor classifier (Scrublet v 0.2.3) [86]. Batch effects were corrected using SciV. The counts were normalized to median total counts, log transformed, and PCA-clustered. Highly variable genes were identified using Seurat (v.5, [87]). Nearest-neighbor clustering was performed using Leiden. Cell annotation followed best practices [88], and they were manually validated using PanglaoDB (accessed 07/2025, [89]). Differential gene expression was performedusing pseudobulk correcting for multiple hypothesis using moderated estimation in DESEQ2 [90]. The pseudotime analysis was performed in Monocle 3 [44], with trajectories examined using *Krt8+* club cells as root.

### 4.9. Mouse Angiopoietin-like 4 ELISA

The BALF collected from the lungs was diluted two-fold before the assay that was performed according to the manufacturer’s instructions (ab210577, Abcam, Waltham, MA, USA). Absorbance was measured using an Infinite 200 Pro (Tecan, Männedorf, Switzerland) with a 450 nm filter. The results were calculated using a line of fit with an R^2^ value > 99% compared to the total amount of BALF collected from each animal.

### 4.10. Lipoprotein Lipase (LPL) Activity Assay

The BALF collected from the lungs was diluted two-fold before performing an LPL fluorogenic assay as per the manufacturer’s instructions (STA-610, Cell Biolabs, Inc., San Diego, CA, USA). Fluorescence was measured using an Infinite 200 Pro (Tecan, Männedorf, Switzerland) with a 485/525 nm filter. The results were calculated using a line of fit with an R2 value > 98% compared to the total amount of BALF collected from each animal.

### 4.11. Statistical Analysis

A two-way Analysis of Variance (ANOVA) with Tukey’s post hoc test was utilized for the analyses of mouse groups. All other data were analyzed using Student’s *t*-test (to compare two conditions) or one-way ANOVA with Sidák multiple comparison. Kaplan–Meyer curve was analyzed using log-rank (Mantel–Cox). For all analyses, *p* < 0.05 was considered statistically significant.

## 5. Conclusions

In this study, we have established and analyzed a model of RSV-PVLD relevant to chronic airway sequelae in childhood RSV LRTI. We identified major features of epithelial atypia, cell state change, dysregulation of clock genes, and implicate ANGPTL4 signaling originating from an activated pericyte population. We conclude that the innate signaling in the *Scgb1a1+* progenitor mediates alveolar atypia by disrupting mesenchymal–epithelial intercellular communication in RSV-PVLD, involving the ANGPTL4 pathway. Overall, these data suggest that RSV-PVLD remodels the airway, shaping the mesenchymal–epithelial trophic niches, and may alter subsequent innate responses.

## Data Availability

The scRNA-seq data are available via the Gene Expression Omnibus under the accession number GSE291797.

## Supplementary Materials

The following supporting information can be downloaded at: https://www.mdpi.com/article/10.3390/ijms27062864/s1.

## Abbreviations

The following abbreviations are used in this manuscript:

ANGPTL4	angiopoietin-like 4
AQP3	aquaporin 3
AT	alveolar type
BMAL	basic helix-loop-helix ARNT-like 1
CKO	conditional knockout
Itg	integrin
Nr1d	nuclear receptor subfamily 1 group D
mTmG	membrane-localized tdTomato GFP mouse
PVLD	post-viral lung disease
RSV	respiratory syncytial virus
scRNA-seq	single-cell RNA-seq
UMAP	Uniform Manifold Approximation and Projection
TRP63	tumor protein 63
Scgb1a1	secretoglobin
