# Supplementary material for: RelA Signaling in Scgb1a1+ Progenitors Mediates Lower Airway Epithelial Atypia in RSV-Induced Post-Viral Lung Disease
Source: Int J Mol Sci. 2026 Mar 21;27(6):2864. doi: 10.3390/ijms27062864 (PMC13026159; doi:10.3390/ijms27062864)

Supplementary Table S1. Primer sequences used.

Name	Primer Sequence (mouse)
gadph	Forward: CAGATCCACGACGGACACATTGGG
	Reverse: CATGACAACTTTGGCATTGTGG
rela	Forward: AAGAGCAGCGTGGGGACTAC
	Reverse: TGCCAGAGTTTCGGTTCACT
aqp3	Forward: TTTGGCTTCGCTGTCACCCTTG
	Reverse: CCAGTGCATAGATGGGCAGCTT
trp63	Forward: GTATCGGACAGCGCAAAGAACG
	Reverse: CTGGTAGGTACAGCAGCTCATC
il33	Forward: CTACTGCATGAGACTCCGTTCTG
	Reverse: AGAATCCCGTGGATAGGCAGAG
**Name**	**Primer Sequence (RSV)**
RSV-F	Forward: GGCAACCAACAATCGAGCCA
	Reverse: CACTGGCGATTGCAGATCCA
Positive sense RNA-specific cDNA synthesis	CGGTCATGGTGGCGAATAATCCTGCAAAAATCCCTTCAACT
Negative sense RNA-specific cDNA synthesis	CGGTCATGGTGGCGAATAAACTTTATAGATGTTTTTGTTCA
Positive sense-specific qPCR primer	CCCCACTTTATAGATGTTTTTGTTCA
Negative sense-specific qPCR primer	TCCTGCAAAAATCCCTTCAACT
qPCR tag primer	CGGTCATGGTGGCGAATAA

Supplementary Table S2. Antibodies used.

Antibody	Dilution Factor	Cat #	Company
Aqp3	1:100_rb	AQP-003	ThermoFisher Scientific
CD68	1:100_rb	ab125212	Abcam
Integrin β4 (CD104)	1:100_mo	sc-514426	Santa Cruz Biotechnology
Nr1d1	1:200_rb	14506-1-AP	Proteintech
p63 (CKAP4)	1:500_rb	CL594-16686	Proteintech
RSV	1:300_gt	ab20745	Abcam
SFTPC	1:300_rb	PA5-71680	ThermoFisher Scientific
ANGTPL4	1:200_rb	40-9800	ThermoFisher Scientific
HIGD1B	1:100_PE Conjugated	bs-15489r	Bioss

Supplementary Table S3. Numbers of cells captured in scRNA seq.

Group	Sample Name	Cells Captured
Control	201	9,122
Control	202	6,656
Control	203	8,683
Control	204	8,008
RSV	221	9,735
RSV	222	8,077
RSV	223	8,188
RSV	225	8,943
RSV+RELA KD	231	8,663
RSV+RELA KD	232	10,243
RSV+RELA KD	233	8,648
RSV+RELA KD	236	9,089

Supplementary Table 4. Fractions of reads in cells (Percent).

Group	Sample Name	Fractions of reads in cells (%)
Control	201	93.56
Control	202	93
Control	203	92.49
Control	204	90.46
RSV	221	93.2
RSV	222	92.94
RSV	223	92.39
RSV	225	93.34
RSV+RELA KD	231	92.4
RSV+RELA KD	232	93.03
RSV+RELA KD	233	93.21
RSV+RELA KD	236	93.15

Supplementary Figure S1. Stratification of O_2 Sats by sex. Resting oxygen saturation was measured by collar oximeter (MouseOx® Plus, Starr Life Sciences Corp., Oakmont, PA). O2 saturations (%) are plotted for each sex as a function of treatment group. Individual symbols are independent mice. Boxes are 25-75% IQR. Left, males; right females.

Supplementary Figure S2. Changes in Itg expression. Quantification of ITGa6 and ITGb4 in mouse lung by immunofluorescence staining. AU, arbitrary fluorescence units. Each symbol is average of multiple images for each individual animal (n=5). **, P<0.01.

Supplementary Figure S3. Uniform Manifold Approximation and Projection (UMAP) of sc-RNA-seq data. (A) representation of single cell RNA sequencing of epithelial mesenchymal cell populations separately from Mock-infected, vehicle-treated, RSV-infected, vehicle-treated or RSV-infected TMX-treated animals harvested 21 d after infection. Each symbol represents single cell. Note the similar patterns of cells for each treatment type. (B) UMAP of individual biological replicates for the entire sample set. Note that the same cell clusters are represented by each biological replicate, indicating minimal sample variation.

Supplementary Figure S4. Heterogeneity of AT2 population. (A) Leiden UMAP clustering of aAT2 cells. Note the presence of transcriptionally distinct subsets of AT2 cells without distinct borders. (B). Leiden clusters highly express surfactant C. Shown is relative

expression of Sftpc mRNA overlaid on the UMAP representation. Note the high Sftpc expression by all the leiden clusters #1, 2, 3, 5, 8, 19 and 20, confirming AT2 lineage. (C). Highest expressing marker genes for each cluster. Shown for each leiden cluster is a rank-order listing of the highest differentially expressed gene. Note Sparc expression in cluster #5, and Scd1 expression in Cluster #8. (D) Sparc mRNA expression overlaid on the parent UMAP. Note the enrichment over cluster 5. (E) Scd1 expression overlaid on the parent UMAP with enrichment in cluster 8. (F) Changes in cell expression as a function of treatment. For each leiden cluster the frequency of cells normalized to total cells was calculated. Note the shift in expression from differentiated AT2 cells (cluster 1) to aAT2 population 2, 8.

Supplementary Figure S1.

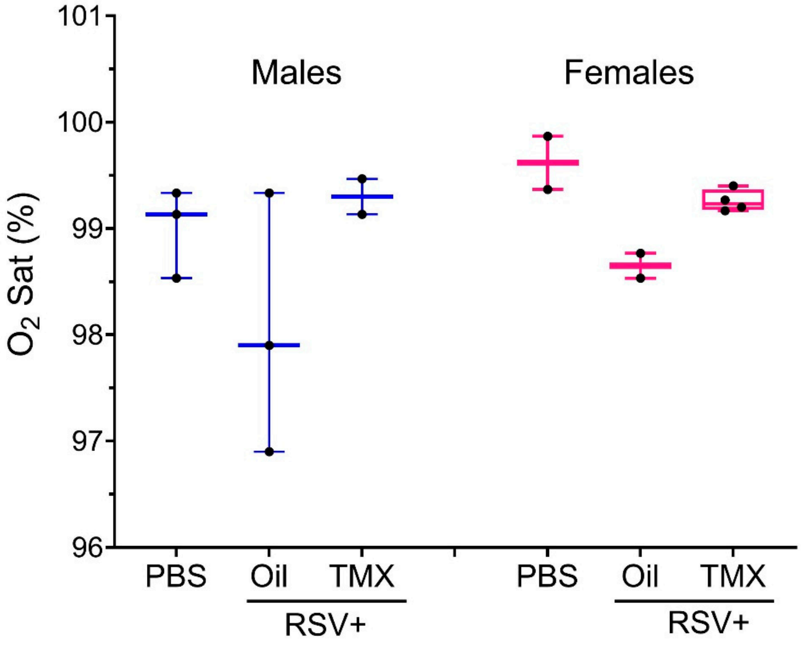

Supplementary Figure S2.

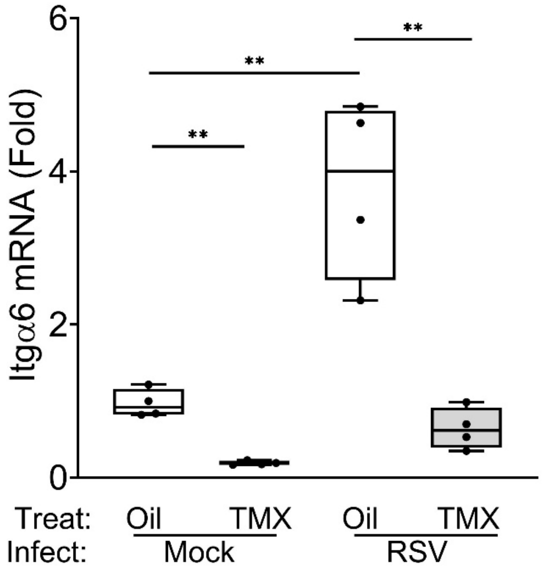

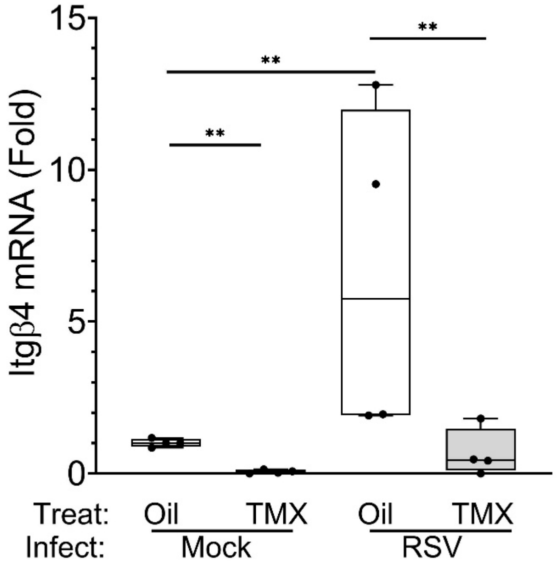

Supplementary Figure S3.

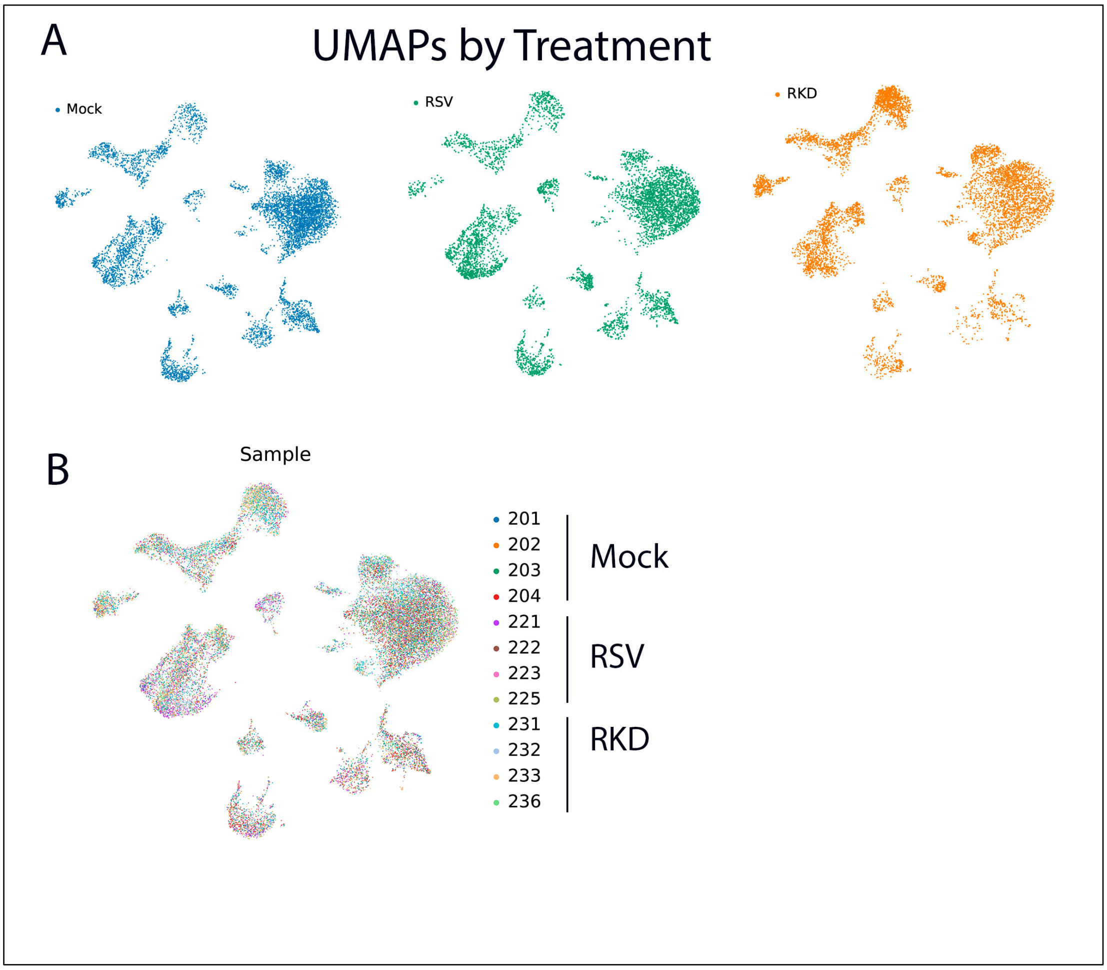

Supplementary Figure S4.

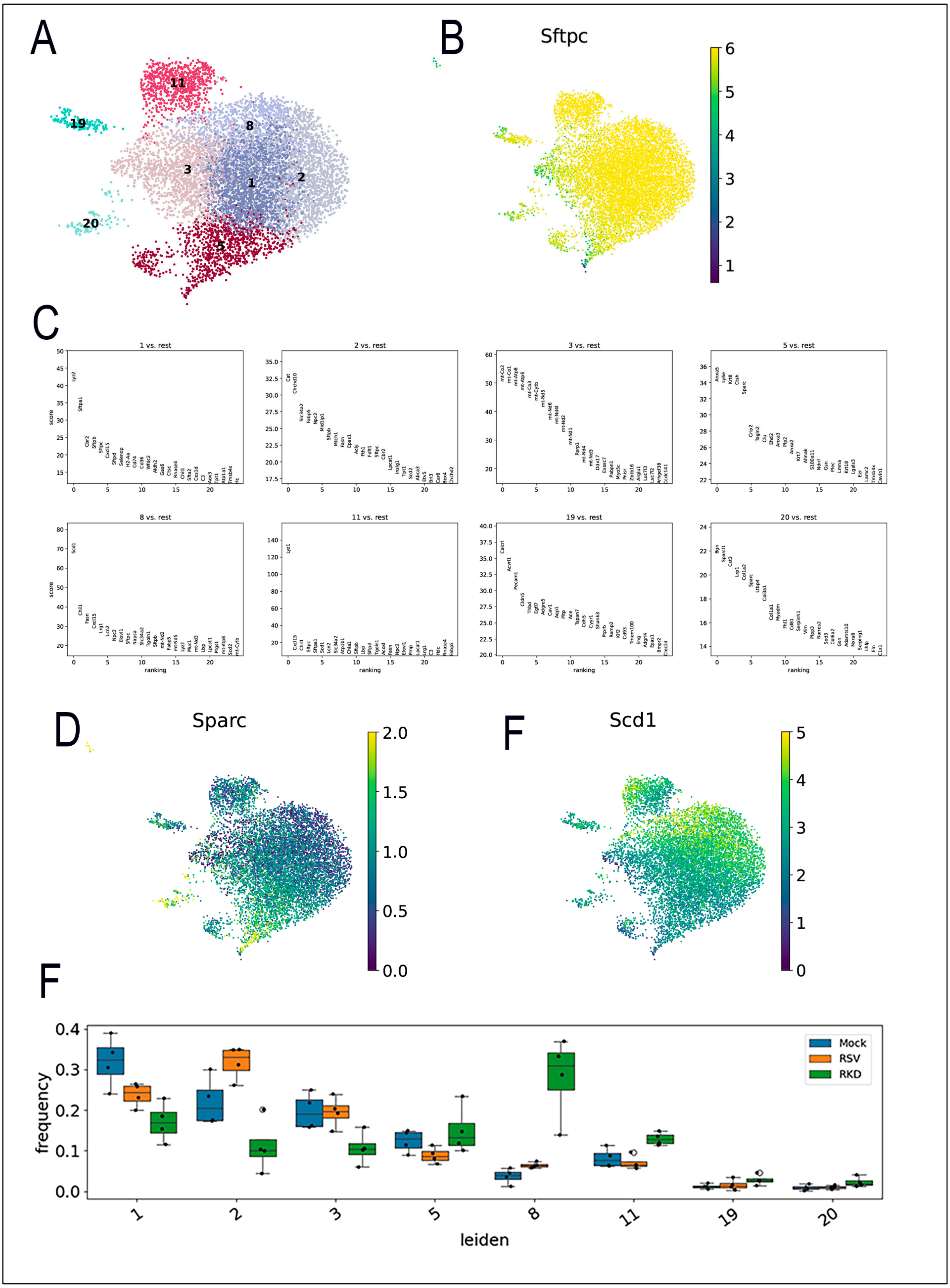

Supplement: Supplementary file 1 [file ijms-27-02864-s001.zip › ijms-4026293-supplementary.pdf]
